# Singular optics empowered by engineered optical materials

**DOI:** 10.1515/nanoph-2023-0030

**Published:** 2023-04-05

**Authors:** Hooman Barati Sedeh, Natalia M. Litchinitser

**Affiliations:** Department of Electrical and Computer Engineering, Duke University, 27708 Durham, NC, USA

**Keywords:** light-matter interaction, Mie resonance, Mie-tronics, optical anapole, singular optics, structured light

## Abstract

The rapid development of optical technologies, such as optical manipulation, data processing, sensing, microscopy, and communications, necessitates new degrees of freedom to sculpt optical beams in space and time beyond conventionally used spatially homogenous amplitude, phase, and polarization. Structuring light in space and time has been indeed shown to open new opportunities for both applied and fundamental science of light. Rapid progress in nanophotonics has opened up new ways of “engineering” ultra-compact, versatile optical nanostructures, such as optical two-dimensional metasurfaces or three-dimensional metamaterials that facilitate new ways of optical beam shaping and manipulation. Here, we review recent progress in the field of structured light–matter interactions with a focus on all-dielectric nanostructures. First, we introduce the concept of singular optics and then discuss several other families of spatially and temporally structured light beams. Next, we summarize recent progress in the design and optimization of photonic platforms, and then we outline some new phenomena enabled by the synergy of structured light and structured materials. Finally, we outline promising directions for applications of structured light beams and their interactions with engineered nanostructures.

## 1 Introduction

Despite the long history of optics, it remains a dynamic research field with an expanding range of applications. It is noteworthy that until the 1990s, optics primarily dealt with plane waves, Gaussian beams, and smooth wavefronts [1]. This situation drastically changed thanks to the pioneering works of Marat Soskin of Ukraine [2–4] and Les Allen of the UK [5–7] who introduced the concepts of experimental “singular optics” and “orbital angular momentum of light”, respectively. Since then, the concepts of singular beams, optical vortices, structured light (also known as sculpted, custom, customized, tailored, and complex light beams) permanently entered the domain of modern optics. Nowadays, it is well-established that a beam of light can carry both spin and orbital angular momenta (SAM and OAM) parallel to the beam axis [8]. In particular, the orbital angular momentum is related to the helical phase front of the light beam, while the spin angular momentum is associated with its polarization [5, 9]. Today, the term “structured light” describes a variety of optical waveforms with the spatial inhomogeneity of one or more physical parameters in two- or three-dimensional space and time, including beams carrying SAM and OAM, Bessel–Gaussian beams [10, 11], radially and azimuthally polarized vector beams [12], optical links and knots [13–15], spatio-temporal optical vortices (STOVs) [16–18], and flying donut pulses [19–21]. Some of these beams already find applications in particle manipulation, optical communications, quantum information processing, sensing, and microscopy [22–25], while others remain the subjects of scientific curiosity primarily because of the practical challenges associated with their experimental realization. It is worth mentioning that several intricate optical structures, such optical knots and skyrmions, have only been achieved in recent years, reflecting the rapid progress and ongoing innovation within this field of research [26–28].

**Figure 1:**
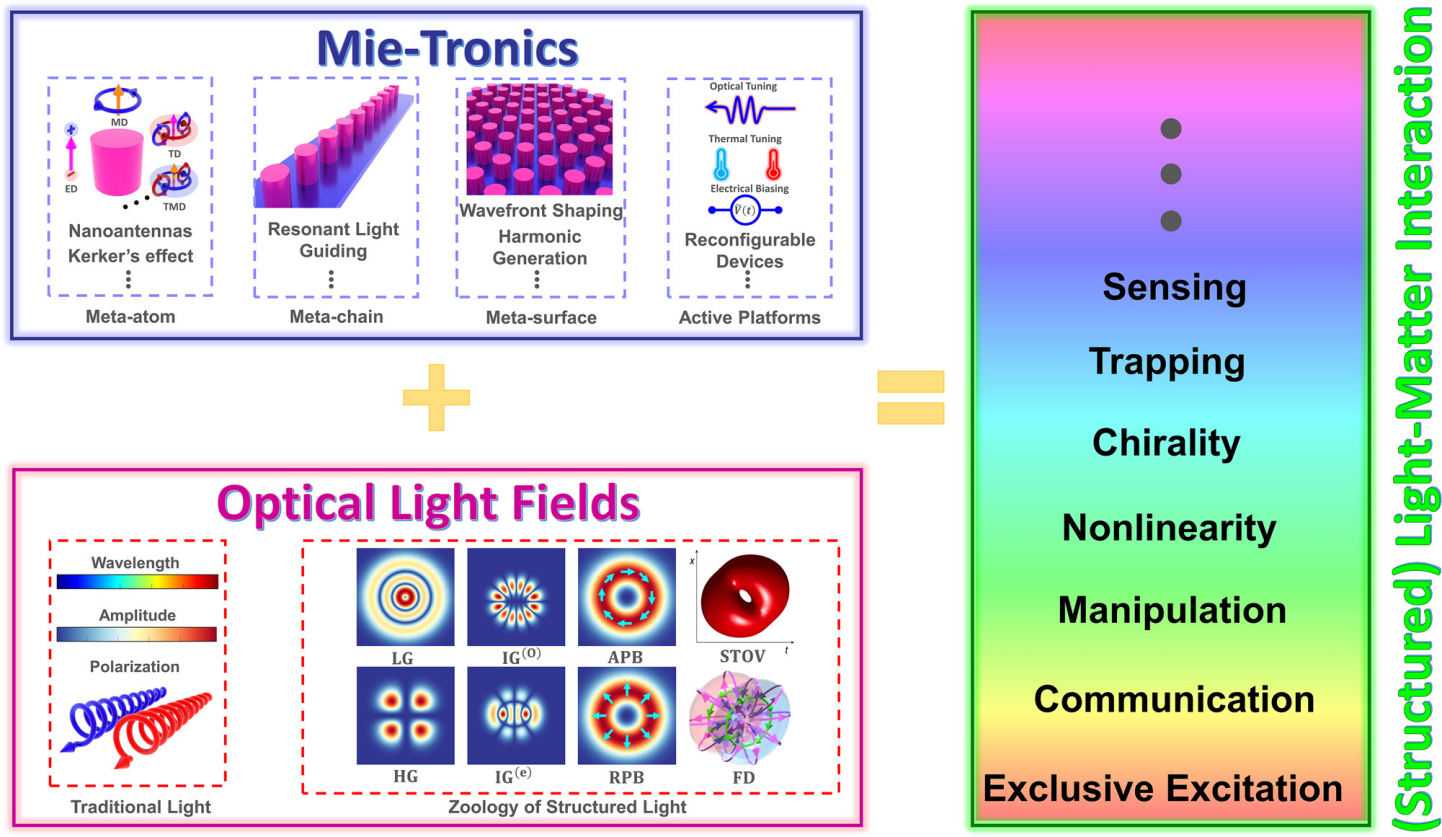
(Top row) The bird’s eye view of main categories of Mie-tronics, ranging from passive structures including single resonator (meta-atom), one dimensional (meta-chain), and two dimensional (meta-surface) periodic resonators as well as possible tuning mechanisms for generating active and reconfigurable platforms. (Bottom row) The zoology of structured lights comprises of various types of light beams ranging from LG beams to flying donut pulses. The combination of these two seemingly unrelated fields leads to unique phenomena such as selective excitation of induced multipolar moments, and nonlinear harmonic generation. The STOV and FD are adapted from Ref [17, 23].

Rapid progress in nanofabrication and computational electromagnetics has opened up new prospects for the realization of versatile optical nanostructures, such as optical two-dimensional metasurfaces [29–31] or three-dimensional metamaterials [32–34] that facilitate unprecedented opportunities for tailoring optical waveforms. For instance, revolutionary developments in computational optics enabled powerful Maxwell’s equation solvers that facilitated the generalization of classical Mie theory first introduced more than a century ago for spherical particles [35] arbitrary shaped complex particles. In parallel, recent progress in electron beam lithography and focused ion beam lithography has enabled the experimental realization of arbitrary shaped particles at nanoscale. These scatterers can be arranged in isolated, two- and three-dimensional arrangements, which are referred to as meta-atoms, metasurfaces, and metamaterials, respectively. It should be remarked that the prefix “meta” means “beyond” in Greek, reflecting that these engineered nanostructures enable optical properties beyond those realizable using conventional (non-structured) materials. Owing to the success and various breakthroughs enabled by such engineered (dielectric) materials, nowadays the unique name of *Mie-tronics* has been dedicated to this field of research [36–39] (see the top row in Figure 1 for the main categories of Mie-tronics). Consequently, Mie-tronics offers exceptional opportunities to design ultra-compact, multifunctional, and reconfigurable components that facilitate structured light generation, detection, shaping, steering, and multiplexing, among various other applications [38–47]. While to date, the research in the field of Mie-tronics was largely limited to conventional Gaussian light beams, in this paper, we aim at reviewing the new phenomena and potential applications that emerge from the synergy of these two branches of optical science – structured light and Mie-tronics. We will briefly review several families of structured light beams and discuss their amplitude, phase, and polarization properties. Next, we review the basic results of Mie theory and discuss both the exact and long wavelength approximation (LWA) multipole decompositions. In the third section, we discuss recent progress in the field of conventional (Gaussian illumination-based) Mie-tronics. Afterward, we provide an overview of recent developments in the field of structured light-structured matter interactions and discuss the potential and unique opportunities enabled by such a synergy. Finally, we provide an outlook for the outstanding opportunities in this rapidly developing field.

## 2 Structured light beams

Contrary to conventional light beams such as plane waves or Gaussian beams, structured light beams are complex optical fields with unique spatial and temporal distributions of amplitude, phase, and/or polarizations [48–54]. Several experimental techniques including a pair of cylindrical lenses [5], spiral phase plates [55, 56] q-plates [57–59], spatial light modulators (SLM) [60–62], and optical metasurfaces [63–69] have been developed to tailor the amplitude, wavefront, and polarization of light for numerous applications such as optical communications, quantum information processing, optical imaging, metrology, microscopy, and optical manipulation [22–25]. Here, we briefly review the mathematical description of structured light beams and highlight their unique features. More details on this subject can be found in [12, 23, 24, 49, 50, 64].

### 2.1 Family of paraxial wave solutions

**Figure 2:**
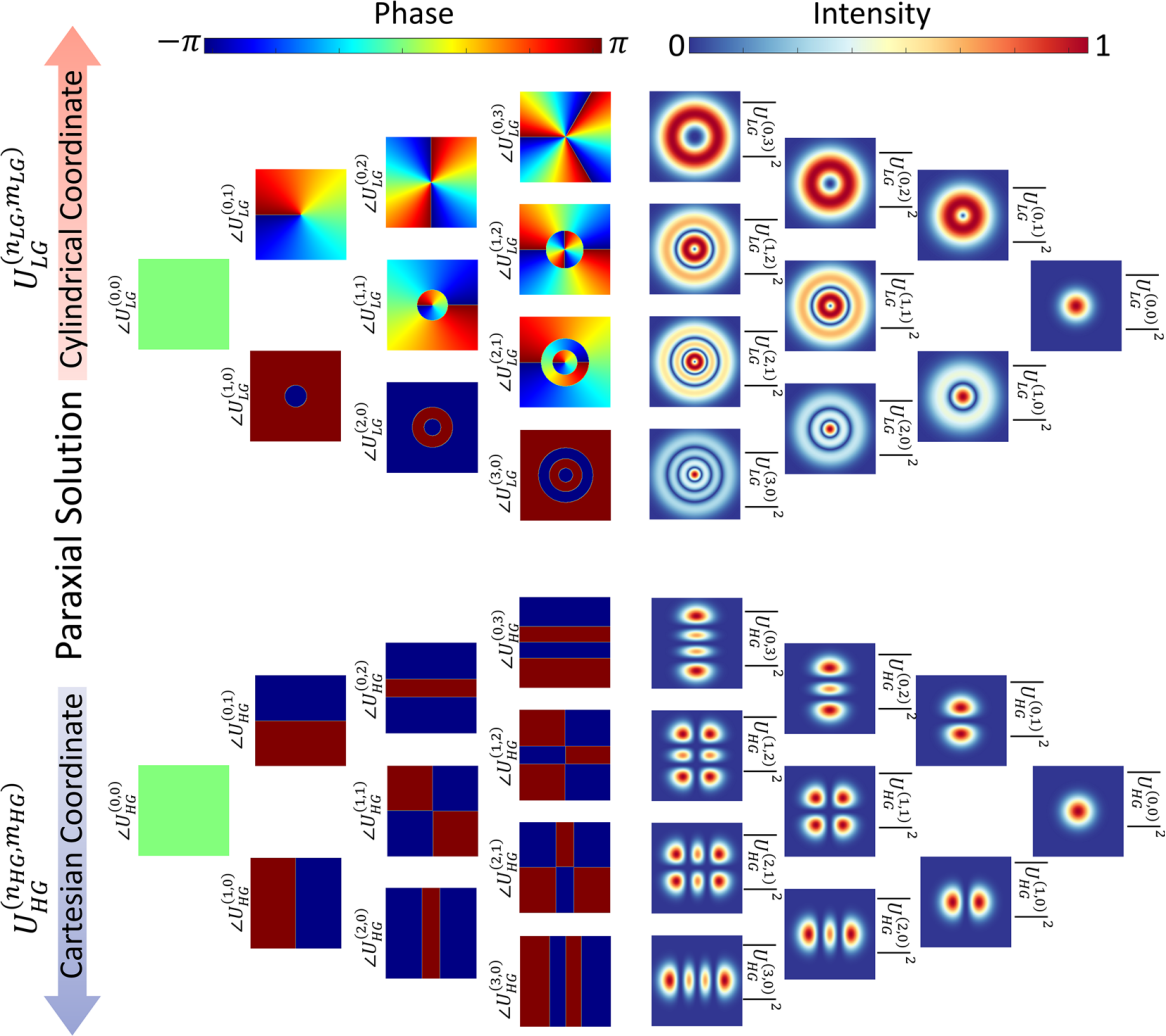
Laguerre–Gaussian and Hermite–Gaussian mode intensity and phase profiles for various combinations of order indices. Apart from the fundamental mode, with $\left(m_{h},n_{h}\right)=\left(p,m\right)=0$ , the corresponding intensity and phase distributions of HG and LG beams differ significantly from Gaussian light beams.

As the starting point, we consider the paraxial solution of the Helmholtz equation which can be expressed as $E\left(r\right)=E_{0}U\left(r\right)\exp\left(-\text{i}\left(kz-\omega t\right)\right)$ , wherein *k* = 2*π*/*λ* is the wavenumber, *ω* is the angular frequency, ***E***_0_ is a complex constant vector and $U\left(r\right)$ represents a complex scalar field, satisfying slowly varying envelope approximation in the *z*-direction as $\left|\frac{\partial^{2}U\left(r\right)}{\partial z^{2}}\right|\ll k^{2}\left|U\left(r\right)\right|$ and $\left|\frac{\partial^{2}U\left(r\right)}{\partial z^{2}}\right|\ll k\left|\frac{\partial U\left(r\right)}{\partial z}\right|$ . One of the most well-known solutions to the paraxial wave equation, $\partial^{2}U\left(r\right)/\partial x^{2}+\partial^{2}U\left(r\right)/\partial y^{2}+\text{i}2k\partial U\left(r\right)/\partial z=0$ , is Gaussian beam that can be written as

(1) $$\begin{array}{ll} U_{\text{G}}\left(r\right) & =\frac{1}{\sqrt{1+{\left(\frac{z}{z_{R}}\right)}^{2}}}\exp\left[-ik\left(x^{2}+y^{2}\right)/2R\left(z\right)\right]\\ & \;\times \exp\left[-\left(x^{2}+y^{2}\right)/w^{2}\left(z\right)\right]\exp\left(i\Phi \left(z\right)\right) \end{array}$$

where $w\left(z\right)=w_{0}\sqrt{1+{\left(z/z_{R}\right)}^{2}}$ is the beam size at a coordinate *z* with *w*_0_ being the beam waist, $R\left(z\right)=\left(z^{2}+z_{R}^{2}\right)/z$ is the radius of the curvature, $\Phi \left(z\right)=\tan^{-1}\left(z/z_{R}\right)$ is the Gouy phase, and $z_{R}=\pi w_{0}^{2}/\lambda$ corresponds to the Rayleigh range [70, 71]. Besides the solution given in Eq. (1), other shape-invariant orthogonal solutions can be found. In particular, in Cartesian coordinates, the so-called Hermite–Gaussian (HG) beams can be obtained as

(2) $$\begin{array}{ll} U_{\text{HG}}^{\left(n_{\text{HG}},m_{\text{HG}}\right)}\left(r\right) & =\sqrt{\frac{2^{\left(1-n_{\text{HG}}-m_{\text{HG}}\right)}}{\pi w_{0}^{2}n_{\text{HG}}!m_{\text{HG}}!}}H_{n_{\text{HG}}}\left(\sqrt{2}\frac{x}{w\left(z\right)}\right)\\ & \;\times H_{m_{\text{HG}}}\left(\sqrt{2}\frac{y}{w\left(z\right)}\right)\\ & \;\times \exp\left[i\left(m_{\text{HG}}+n_{\text{HG}}\right)\Phi \left(z\right)\right]\cdot U_{\text{G}}\left(r\right) \end{array}$$

where $H_{\alpha }\left(\gamma \right)$ are the Hermite polynomials of order *α*, with *m*_HG_ and *n*_HG_ represent the HG mode indices [72]. On the other hand, in the cylindrical coordinate system, $r=\sqrt{x^{2}+y^{2}}$ , $\varphi =atan\left(y/x\right)$ , and *z* = *z*, the paraxial solution leads to the so-called Laguerre–Gaussian (LG) modes, expressed as

(3) $$\begin{array}{ll} U_{\text{LG}}^{\left(n_{\text{LG}},m_{\text{LG}}\right)}\left(r\right) & =\sqrt{\frac{2n_{\text{LG}}!}{\pi \left(n_{\text{LG}}+\left|m_{\text{LG}}\right|\right)!}}{\left(\frac{\sqrt{2}r}{w\left(z\right)}\right)}^{\left|m_{\text{LG}}\right|}\\ & \;\times L_{n_{\text{LG}}}^{\left|m_{\text{LG}}\right|}\left(\frac{2r^{2}}{w^{2}\left(z\right)}\right)\exp\left[i\left(\left|m_{\text{LG}}\right|\right.\right.\\ & \;\left.\left.+2n_{\text{LG}}\right)\Phi \left(z\right)\right]\exp\left(-im_{\text{LG}}\varphi \right)\cdot U_{\text{G}}\left(r\right) \end{array}$$

where *n*_LG_ and *m*_LG_ represent the radial and azimuthal mode indices of the LG beam, and $L_{n_{\text{LG}}}^{\left|m_{\text{LG}}\right|}\left(\gamma \right)$ denotes the Laguerre polynomials [72]. When comparing Eq. (3) with Eq. (1), it becomes evident that for *m*_LG_ ≠ 0, the structure of the LG beam differs from that of the regular Gaussian beam. This is due to an additional phase term of exp(−*im*_LG_ *φ*), which results in a phase singularity along the beam axis and a dark central spot in its spatial cross-section. To further illustrate the differences between the HG and LG structured light beams and the conventional Gaussian beam, in Figure 2 we provide their corresponding intensity and phase distributions. As can be seen from Figure 2, when the corresponding indices (*n*_HG_, *m*_HG_) or (*n*_LG_, *m*_LG_) are zero, both HG and LG paraxial solutions reduce to the fundamental Gaussian beam. However, once the mode indices acquire nonzero values, their corresponding intensity and phase distributions differ significantly from one another. Note that the differences in the intensity distributions are not the only, and in fact, not the most important differences between the LG/HG and Gaussian beams. In particular, the phase distributions of these two beams (i.e., LG/HG) differ drastically from the Gaussian light beams, with discontinuities and singularities associated with the structure of the given mode. However, despite the differences between the LG and HG light beams, on account of the underlying symmetry between these families of modes, they can be related to one another [73, 74]. To show such a conversion between these two modes, we start by rewriting the LG expression in terms of even and odd modes instead of exponentials as follow:

(4) $$\begin{array}{ll} \left\{\begin{array}{c} U_{\text{even}}^{\left(n_{\text{LG}},m_{\text{LG}}\right)}\\ U_{\text{odd}}^{\left(n_{\text{LG}},m_{\text{LG}}\right)} \end{array}\right\} & =\sqrt{\frac{2n_{\text{LG}}!}{\pi \left(n_{\text{LG}}+\left|m_{\text{LG}}\right|\right)!}}{\left(\frac{\sqrt{2}r}{w\left(z\right)}\right)}^{\left|m_{\text{LG}}\right|}\\ & \;\times L_{n_{\text{LG}}}^{\left|m_{\text{LG}}\right|}\left(\frac{2r^{2}}{w^{2}\left(z\right)}\right)\exp\left[i\left(\left|m_{\text{LG}}\right|\right.\right.\\ & \;\left.\left.\times +2n_{\text{LG}}\right)\Phi \left(z\right)\right]\cdot U_{\text{G}}\left(r\right)\left\{\begin{array}{c} \cos\left(m_{\text{LG}}\varphi \right)\\ \sin\left(m_{\text{LG}}\varphi \right) \end{array}\right\} \end{array}$$

The relation between the LG and HG modes are obtained based expressing the $\cos\left(m_{\text{LG}}\varphi \right)r^{m_{\text{LG}}}L_{n_{\text{LG}}}^{\left|m_{\text{LG}}\right|}\left(r^{2}\right)$ and $\sin\left(m_{\text{LG}}\varphi \right)r^{m_{\text{LG}}}L_{n_{\text{LG}}}^{\left|m_{\text{LG}}\right|}\left(r^{2}\right)$ in terms of Hermite polynomials of $H_{\alpha }\left(x\right)$ and $H_{\beta }\left(y\right)$ as (the interested reader is referred to [73] for the mathematical details)

(5) $$\begin{array}{cc} & \cos\left(m_{\text{LG}}\varphi \right)r^{m_{\text{LG}}}L_{n_{\text{LG}}}^{\left|m_{\text{LG}}\right|}\left(r^{2}\right)\\ & \;=\frac{{\left(-1\right)}^{n_{\text{LG}}}}{2^{2n_{\text{LG}}+m_{\text{LG}}}n_{\text{LG}}!}\overset{n_{\text{LG}}}{\underset{l=0}{\sum }}\overset{\left[\frac{m_{\text{LG}}}{2}\right]}{\underset{s=0}{\sum }}{\left(-1\right)}^{s}\left(\begin{array}{c} n_{\text{LG}}\\ l \end{array}\right)\\ & \;\;\times \left(\begin{array}{c} m_{\text{LG}}\\ 2s \end{array}\right)H_{2\left(l-s\right)+m_{\text{LG}}}\left(x\right)H_{2\left(n_{\text{LG}}-l+s\right)}\left(y\right)\\ & \sin\left(m_{\text{LG}}\varphi \right)r^{m_{\text{LG}}}L_{n_{\text{LG}}}^{\left|m_{\text{LG}}\right|}\left(r^{2}\right)\\ & \;=\frac{{\left(-1\right)}^{n_{\text{LG}}}}{2^{2n_{\text{LG}}+m_{\text{LG}}}n_{\text{LG}}!}\overset{n_{\text{LG}}}{\underset{l=0}{\sum }}\overset{\left[\left(m_{\text{LG}}-1\right)/2\right]}{\underset{s=0}{\sum }}{\left(-1\right)}^{s}\left(\begin{array}{c} n_{\text{LG}}\\ l \end{array}\right)\\ & \;\;\times \left(\begin{array}{c} m_{\text{LG}}\\ 2s+1 \end{array}\right)H_{2\left(l-s\right)+m_{\text{LG}}-1}\left(x\right)H_{2\left(n_{\text{LG}}-l+s\right)+1}\left(y\right) \end{array}$$

By inserting Eq. (5) into Eq. (4), the LG of even and odd modes can be represented in terms of HG light beam as

(6) $$\begin{array}{cc} U_{\text{even}}^{\left(n_{\text{LG}},m_{\text{LG}}\right)} & ={\left(-1\right)}^{n_{\text{LG}}}\overset{n_{\text{LG}}}{\underset{l=0}{\sum }}\overset{\left[\frac{m_{\text{LG}}}{2}\right]}{\underset{s=0}{\sum }}\\ & \;\times \sqrt{\frac{\left[2\left(l-s\right)+m_{\text{LG}}\right]!\left[2\left(n_{\text{LG}}-l+s\right)\right]!}{2^{2n_{\text{LG}}+m_{\text{LG}}-1}n_{\text{LG}}!\left(n_{\text{LG}}+\left|m_{\text{LG}}\right|\right)!}}\\ & \;\times {\left(-1\right)}^{s}\left(\begin{array}{c} n_{\text{LG}}\\ l \end{array}\right)\left(\begin{array}{c} m_{\text{LG}}\\ 2s \end{array}\right)\cdot U_{\text{HG}}^{\left(2\left(l-s\right)+m_{\text{LG}},2\left(n_{\text{LG}}-l+s\right)\right)}\\ U_{\text{odd}}^{\left(n_{\text{LG}},m_{\text{LG}}\right)} & ={\left(-1\right)}^{n_{LG}}\overset{n_{LG}}{\underset{l=0}{\sum }}\overset{\left[\left(m_{\text{LG}}-1\right)/2\right]}{\underset{s=0}{\sum }}\\ & \;\times \sqrt{\frac{\left[2\left(l-s\right)+m_{\text{LG}}-1\right]!\left[2\left(n_{\text{LG}}-l+s\right)+1\right]!}{2^{2n_{\text{LG}}+m_{\text{LG}}-1}n_{\text{LG}}!\left(n_{\text{LG}}+\left|m_{\text{LG}}\right|\right)!}}\\ & \;\times {\left(-1\right)}^{s}\left(\begin{array}{c} n_{\text{LG}}\\ l \end{array}\right)\left(\begin{array}{c} m_{\text{LG}}\\ 2s+1 \end{array}\right)\\ & \;\cdot U_{\text{HG}}^{\left(2\left(l-s\right)+m_{\text{LG}}-1,2\left(n_{\text{LG}}-l+s\right)+1\right)} \end{array}$$

As can be seen from Eq. (6), the LG light beam of even and odd modes can be represented as the superposition of HG beams with various mode indices. Specifically, even modes can be expressed using a combination of $\left(n_{\text{LG}}+1\right)+\left[m_{\text{LG}}/2\right]$ HG beams, whereas the odd mode requires $\left(n_{\text{LG}}+1\right)+\left[\left(m_{\text{LG}}+1\right)/2\right]$ HG beams [73]. We note that the HG mode can also be expressed as the superposition of LG beams, which its mathematical derivations are outlined elsewhere [73, 74].

**Figure 3:**
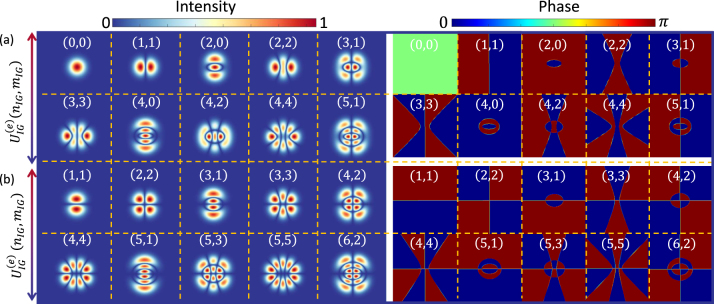
Transverse field distributions of amplitude and phase of (a) even and (b) odd Inc-Gaussian light beams with *ɛ* = 2 and for various combinations of $\left(n_{\text{IG}},m_{\text{IG}}\right)$ indicated in the inset of each plot.

It is noteworthy that while Poincare sphere is commonly used to visualize the polarization of light, it can also be a useful tool for understanding complex polarization states, and other degrees of freedom such as OAM [75]. Similar to its conventional counterpart, such a representation is referred to as higher order Poincare sphere throughout the literature and can provide a more complete understanding of the properties and behavior of electromagnetic waves [76]. In addition, the so-called Ince–Gaussian (IG) beams are known to be the exact analytical solutions of the paraxial wave equation in an elliptic cylindrical coordinates system [77]. The general expression of the IG can be decomposed into even and odd solutions, each written as

(7) $$\begin{array}{c} U_{\text{IG}}^{\left(e\right)}\left(r\right)=C_{n_{\text{IG}}}^{m_{\text{IG}}}\left(i\zeta ,\varepsilon \right)C_{n_{\text{IG}}}^{m_{\text{IG}}}\left(\eta ,\varepsilon \right)\exp\left[ip\Phi \left(z\right)\right]\times U_{\text{G}}\left(r\right)\\ U_{\text{IG}}^{\left(o\right)}\left(r\right)=S_{n_{\text{IG}}}^{m_{\text{IG}}}\left(i\zeta ,\varepsilon \right)S_{n_{\text{IG}}}^{m_{\text{IG}}}\left(\eta ,\varepsilon \right)\exp\left[ip\Phi \left(z\right)\right]\times U_{\text{G}}\left(r\right) \end{array}$$

where *ɛ* indicates the ellipticity parameter, $C_{n_{\text{IG}}}^{m_{\text{IG}}}\left(\text{i}\zeta ,\eta \right)$ and $S_{n_{\text{IG}}}^{m_{\text{IG}}}\left(\text{i}\zeta ,\eta \right)$ are even and odd Ince polynomials of order *n*_IG_ and degree *m*_IG_, such that 1 < *m*_IG_ < *n*_IG_ for odd function, 0 < *m*_IG_ < *n*_IG_ for even function, and the indices $\left(n_{\text{IG}},m_{\text{IG}}\right)$ have the same parity ${\left(-1\right)}^{n_{\text{IG}}-m_{\text{IG}}}=1\;\left[78\right.\;,79]$ . Moreover, $\zeta \in \left[0,\infty \right)$ and $\eta \in \left[0,2\pi \right)$ denote the radial and azimuthal elliptical coordinates that are related to their Cartesian counterparts via $x=\sqrt{\varepsilon /2}w\left(z\right)\cosh\left(\zeta \right)\cos\left(\eta \right)$ and $y=\sqrt{\varepsilon /2}w\left(z\right)\sinh\left(\zeta \right)\sin\left(\eta \right)$ , respectively. Note that the HG and LG beams are special cases of IG beams, when the ellipticity parameter approaches *ɛ* → ∞ and *ɛ* → 0, respectively [72]. Figure 3 shows the intensity and phase distributions of various Ince-Gaussian beam orders.

As can be seen from Figure 3, the beam widths of the $U_{\text{IG}}\left(r\right)$ for higher indices of $\left(n_{\text{IG}},m_{\text{IG}}\right)$ have larger values compared to lower indices. More importantly, the value of *m*_IG_ corresponds to the number of hyperbolic nodal lines, while $\left(n_{\text{IG}}-m_{\text{IG}}\right)/2$ represents the number of elliptic nodal lines [77]. It should be remarked that it is possible to construct the so-called helical IG beam (HIG) from the superposition of Eq. (7) as $U_{\text{HIG}}^{\pm }\left(r\right)=U_{\text{IG}}^{\left(e\right)}\left(r\right)\pm \text{i}U_{\text{IG}}^{\left(o\right)}\left(r\right)$ , which is merely valid for *m*_IG_ > 0 since $U_{\text{IG}}^{\left(o\right)}\left(r\right)$ is undefined for *m*_IG_ = 0 [78]. The helical counterpart of IG beams can be applied to study the transferring of angular momentum to particles, and to construct elliptic optical tweezers and atom traps [74]. As a final remark, we note that the singularities in light beams can arise from two mechanisms of: (i) interference-induced singularities and (ii) topological phase features. In the former scenario, the interference of light beams (typically with specific phase differences) lead to the formation of singularities without an explicit phase structure, whereas in the latter case the singularities are formed due to the presence of a phase singularity or dislocation in the phase, resulting in a helical wavefront structure with an intensity null at the center. However, it should be noted that the singularities arising from topological phase features, tend to be more robust to propagation and are less sensitive to small perturbations, distortions, or noise during propagation.

### 2.2 Cylindrical vector beams

**Figure 4:**
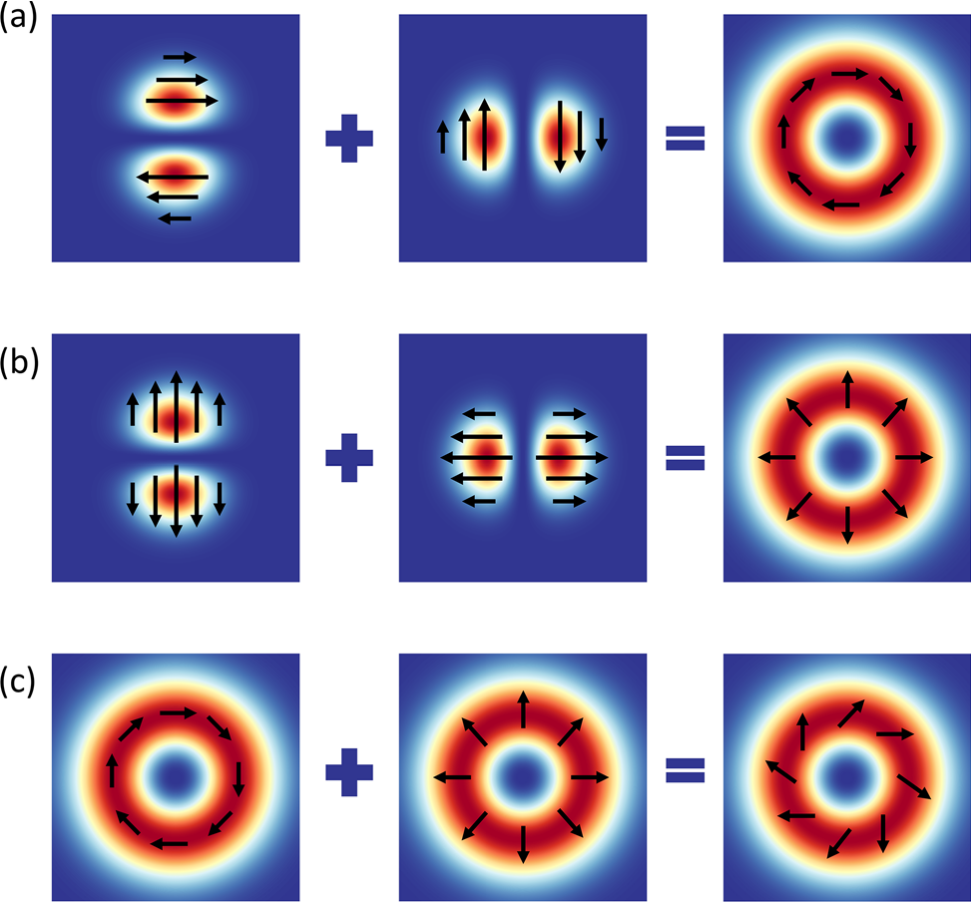
The field distributions of 1st-order CVBs obtained based on the linear superposition of $U_{\text{HG}}^{\left(1,0\right)}\left(r\right)$ and $U_{\text{HG}}^{\left(0,1\right)}\left(r\right)$ complex fields for (a) azimuthally and (b) radially polarized beams. (c) A hybrid CVB can be considered as the superposition of the APB and RPB. The arrows represent the local direction of the polarization vector.

While traditionally the polarization state of light was assumed to be spatially uniform (linear, circular, and elliptical), light beams with spatially varying polarizations are expected to open new avenues to novel phenomena in optical systems. Indeed, the vectorial nature of light beams, their generation, as well as their interaction with matter gained significant attention in the last decade [12]. In particular, cylindrical vector beams (CVBs) are considered to be one special class of such vectorial light beams having cylindrically symmetric inhomogeneous polarizations, which its simplest form (1st-order CVB) can be expressed in terms of the superposition of two $U_{\text{HG}}^{\left(1,0\right)}\left(r\right)$ and $U_{\text{HG}}^{\left(0,1\right)}\left(r\right)$ as [14]

(8) $$\begin{array}{c} E_{\text{APB}}\left(r\right)=U_{\text{HG}}^{\left(0,1\right)}\left(r\right){\hat{e}}_{x}-U_{\text{HG}}^{\left(1,0\right)}\left(r\right){\hat{e}}_{y}\\ E_{\text{RPB}}\left(r\right)=U_{\text{HG}}^{\left(1,0\right)}\left(r\right){\hat{e}}_{x}+U_{\text{HG}}^{\left(0,1\right)}\left(r\right){\hat{e}}_{y} \end{array}$$

where the subscripts of APB and RPB denote the azimuthally and radially polarized beams shown in Figure 4(a) and (b), respectively. We note that the superposition of RPB and APB leads to the so-called hybrid CVB shown in Figure 4(c).

### 2.3 Non-separable states of light

So far, we discussed time-independent solutions of Maxwell’s equations. However, a completely different class of exact solutions to Maxwell’s equations exists, known as non-separable space-time solutions, where the spatial and temporal features of the optical fields are coupled and cannot be separated [48]. The first three-dimensional, non-dispersive, source-free solutions to homogeneous Maxwell’s equations have been introduced by Brittingham and named the focused wave mode (FWM) solutions [79]. However, these first mathematical FWM solutions carried infinite energies. Later, in 1985, Ziolkowski solved the paradox of infinite energy by superposing focus wave modes with carefully chosen weighting functions. These new solutions were called electromagnetic directed energy pulse trains (EDEPTs) [80, 81]. The family of EDEPTs includes non-diffracting pulses with azimuthal dependence [82], focused pancake pulses [83, 84], and Flying Donut (FD) pulses [19, 85]. In particular, FD pulses are characterized by exotic donut-like topological structures and have been shown to provide nontrivial light–matter interaction on account of the similarity between its structural features and the radiation pattern of toroidal dipolar moments [86]. Nevertheless, due to their spatiotemporal structural complexity, the studies of FD pulses have been limited to theory and numerical simulations. However, recently Zheludev et al. proposed and realized for the first time an experimental approach for the generation and propagation of FD pulses using photonic metasurfaces [21]. Another class of structured light beams is the spatiotemporal optical vortex whose phase and energy circulate in both planes of space and time [16, 17]. Thanks to the recent experimental demonstration of STOVs by Milchberg et al. [16, 18, 87], these exotic light beams have gained the attention of the scientific community due to their importance in both theoretical and practical studies such as in describing the particle collisions, optics of moving media and quantum communications [17].

## 3 Light-matter interaction at nanoscale

### 3.1 Mie scattering of nanoparticles

**Figure 5:**
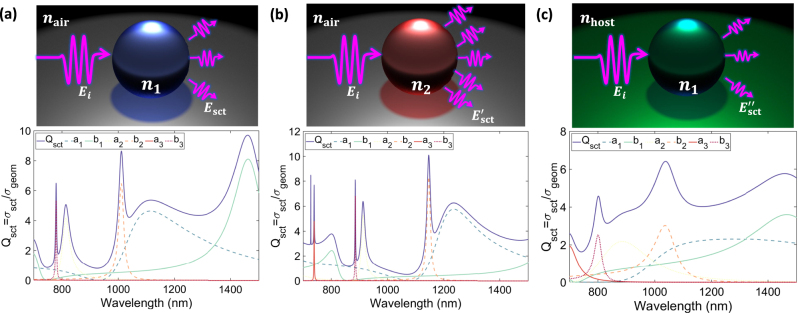
Optical response of a subwavelength scatterer. (a) The scattering response of a dielectric nanosphere derived from Mie theory up to the octupolar terms (*a*_3_ and *b*_3_). (b) The optical behavior of the subwavelength scatterers when its refractive index changes from *n*_1_ = 3.5 to *n*_2_ = 4. (c) The scattering spectrum of the same particle when the host medium refractive index is altered from air to glass.

Light scattering by subwavelength spherical particles can be qualitatively described by Mie theory [35, 88, 89]. According to this theory, the extinction, and scattering efficiencies that are defined as the ratios between their corresponding cross-sections to the geometrical cross-section are given by

(9) $$\begin{array}{c} Q_{\text{ext}}=\frac{\sigma_{\text{ext}}}{\sigma_{\text{geom}}}=\frac{\lambda^{2}}{2\pi R^{2}}\overset{\infty }{\underset{l=1}{\sum }}\left(2l+1\right)Re\left[\left(a_{l}+b_{l}\right)\right]\\ Q_{\text{sct}}=\frac{\sigma_{\text{sct}}}{\sigma_{\text{geom}}}=\frac{\lambda^{2}}{2\pi R^{2}}\overset{\infty }{\underset{l=1}{\sum }}\left(2l+1\right)\left[{\left|a_{l}\right|}^{2}+{\left|b_{l}\right|}^{2}\right] \end{array}$$

where *R* is the radius of the spherical particle, *l* is the orbital mode order ( $l=1,2,3,\ldots \left.\right)$ corresponding to dipolar, quadrupolar, and octupolar contributions, and *a*_*l*_, *b*_*l*_ correspond to the electric and magnetic scattering amplitudes as

(10) $$\begin{array}{c} a_{l}=\frac{n{\psi^{\prime }}_{l}\left(\beta \right)\psi_{l}\left(n\beta \right)-\psi_{l}\left(\beta \right){\psi^{\prime }}_{l}\left(n\beta \right)}{\left(n{\psi^{\prime }}_{l}\left(\beta \right)\psi_{l}\left(n\beta \right)-\psi_{l}\left(\beta \right){\psi^{\prime }}_{l}\left(n\beta \right)\right)+\text{i}\left(n{\chi^{\prime }}_{l}\left(\beta \right)\psi_{l}\left(n\beta \right)-{\psi^{\prime }}_{l}\left(n\beta \right)\chi_{l}\left(\beta \right)\right)},\\ b_{l}=\frac{n{\psi^{\prime }}_{l}\left(n\beta \right)\psi_{l}\left(\beta \right)-\psi_{l}\left(n\beta \right){\psi^{\prime }}_{l}\left(\beta \right)}{\left(n{\psi^{\prime }}_{l}\left(n\beta \right)\psi_{l}\left(\beta \right)-\psi_{l}\left(n\beta \right){\psi^{\prime }}_{l}\left(\beta \right)\right)+\text{i}\left(n\chi_{l}\left(\beta \right){\psi^{\prime }}_{l}\left(n\beta \right)-\psi_{l}\left(n\beta \right){\chi^{\prime }}_{l}\left(\beta \right)\right)}, \end{array}$$

where *β* = 2*πR*/*λ* is the normalized size parameter, *n* is the refractive index of the particle, and $\psi_{l}\left(\beta \right)=\sqrt{\pi \beta /2}J_{l+\frac{1}{2}}\left(\beta \right)$ and $\chi_{l}\left(\beta \right)=\sqrt{\pi \beta /2}N_{l+\frac{1}{2}}\left(\beta \right)$ are auxiliary functions related to Bessel and Neumann functions, respectively [88]. To understand the underlying physics described by Eqs. (9) and (10) and study the effect of different parameters on the optical response of the spherical particle, in Figure 5 we have plotted the scattering response of a dielectric nanosphere as the function of operating wavelength for different scenarios. In particular, Figure 5(a) shows the scattering efficiency (up to octupolar term, *l* = 3) of a dielectric spherical particle, whose radius and refractive index are set to *R* = 200 nm and *n*_1_ = 3.5, respectively. As can be seen from this figure, the optical response of the spherical particle is the superposition of various moments including electric and magnetic dipole (ED; a_1_ and MD; b_1_ respectively) electric and magnetic quadrupole (EQ; a_2_ and MQ; b_2_ respectively) and electric and magnetic octupole (EO; a_3_ and MO; b_3_ respectively). Interestingly, even though the sphere is nonmagnetic, that is its relative permeability is *μ*_r_ = 1, Mie theory predicts the existence of either pure electric or magnetic responses within its scattering spectrum. In Figure 5(b), we have fixed the radius of the spherical particle to *R* = 200 nm and changed its refractive index to *n*_2_ = 4, to evaluate how the refractive index can alter the optical response of subwavelength particles. As can be observed from this panel, changing the refractive index directly alters the scattering response of the particle and results in extra resonant peaks, such as electric octupole (*l* = 3), within its spectrum.

Figure 5(c) demonstrates the scenario wherein the radius and refractive index of the spherical particle are the same as that of Figure 5(a), yet the optical properties of the host medium change from the air (*n*_air_ = 1) to glass ( $n_{\text{host}}=1.5\left.\right)$ . Varying the refractive index of the background medium can also alter the optical response of spherical particle and reduce the magnitude of each peak, which is attributed to the reduction in the contrast between the host medium and particle refractive indices. It should be noted that the original Mie theory has been developed to describe the optical response of spherical particles with any size and materials [35], whereas a theoretical description of light scattering by nonspherical particles has been developed based on the multipole expansion approach [90–94].

### 3.2 Multipole expansion for arbitrary shape meta-atoms: exact moments and long-wavelength approximation

The optical response of nonmagnetic meta-atoms is characterized by the electric current density ***J***_in_ and polarization ***P***_in_ induced by external electromagnetic fields. In the case of monochromatic fields, these values are connected by ***J***_in_ = −i*ω* ***P***_in_, wherein *ω* represents the optical angular frequency [68]. Upon the interaction of light with the meta-atom, the induced polarization is related to the field distributions within the particle via $P_{\text{in}}=\varepsilon_{0}\left(\varepsilon_{p}-\varepsilon_{d}\right)E_{p}$ , where *ɛ*_0_, *ɛ*_*p*_ and *ɛ*_*d*_ are the free space, meta-atom, and surrounding medium dielectric constants, respectively, and ***E***_*p*_ is the total electric field inside the meta-atom. Therefore, the internal field distribution can directly change the induced polarization which in turn alters the excited moments within the meta-atom. In the far-field region, the scattered field can be expressed as [90]

(11) $$\begin{array}{ll} E_{n}\left(r\right) & =i\omega \mu_{0}\frac{\exp\left(ik_{d}r\right)}{4\pi r}\left(\bar{\bar{I}}-nn\right)\int_{V_{s}}J_{\text{in}}\left(r^{\prime }\right)\\ & \;\times \exp\left(-ik_{d}\left(n\cdot r^{\prime }\right)\right)\text{d}r^{\prime } \end{array}$$

where ***n*** = ***r***/*r* is the unit vector directed from the particle’s center towards an observation point, *k*_*d*_ is the wavenumber in the surrounding medium, *μ*_0_ is the permeability of free space, and *I* is a 3 × 3 unitary matrix. The multipole decomposition of scattered waves can be obtained based on three approaches: (a) Taylor expansion of $\exp\left(-\text{i}k_{d}r\right)$ around a point with the radius vector ***r***_0_ located in the meta-atom volume [89]; (b) writing the induced current density within the meta-atom in terms of a Dirac delta as $J_{\text{in}}\left(r^{\prime }\right)=\int_{V_{s}}J_{\text{in}}\left(r\right)\delta \left(r^{\prime }-r\right)\text{d}r^{\prime }$ , and then, performing Taylor expansion of $\delta \left(r^{\prime }-r\right)$ term in the vicinity of point ***r***_0_ [91, 92], and (c) spherical harmonic expansion of $\exp\left(-ik_{d}r\right)$ term directly [90]. As was shown in [90], the first two methods ((a) and (b)) lead to the same results and provide multipoles in the long wavelength approximation (LWA) regime, while the third approach yields the multipole decomposition of the scattered field by finite-size scatterers with arbitrary dimensions providing the exact multipole moments. To obtain the exact moments, the plane wave representation of $\exp\left(-ik_{d}\left(n\cdot r^{\prime }\right)\right)$ in the spherical harmonics leads to

(12) $$\begin{array}{ll} \exp\left(-ik_{d}\left(n\cdot r^{\prime }\right)\right) & =4\pi \overset{\infty }{\underset{l=0}{\sum }}\overset{l}{\underset{m=-l}{\sum }}{\left(-i\right)}^{l}j_{l}\left(k_{d}r^{\prime }\right)Y_{lm}^{*}\\ & \;\times \left(\theta ,\varphi \right)Y_{lm}\left(\theta ,\varphi \right) \end{array}$$

wherein $Y_{lm}\left(\theta ,\varphi \right)$ represents the spherical harmonics, $j_{l}\left(k_{d}r^{\prime }\right)$ is the spherical Bessel function of order *l*, and the asterisk (*) denotes the complex conjugation [89]. Substituting Eq. (12) into Eq. (11), the scattered electric field can be obtained as

(13) $$\begin{array}{ll} E_{n}\left(r\right) & =i\omega \mu_{0}\frac{\exp\left(ik_{d}r\right)}{4\pi r}\left(\bar{\bar{I}}-nn\right)\overset{\infty }{\underset{l=0}{\sum }}{\left(-i\right)}^{l}\left(2l+1\right)\int_{V_{s}}\\ & \;\times J_{\text{in}}\left(r^{\prime }\right)P_{l}\left(\cos\left(\gamma \right)\right)\text{d}r^{\prime } \end{array}$$

where $P_{l}\left(\cos\left(\gamma \right)\right)$ is the *l*-order Legendre polynomial, obtained from the application of the addition theorem for spherical harmonics, and *γ* is the angle between two-unit vectors of $n^{\prime }=r^{\prime }/r^{\prime }=\left(\sin\theta^{\prime }\cos\varphi^{\prime },\sin\theta^{\prime }\sin\varphi^{\prime },\cos\theta^{\prime }\right)$ and $n=r/r=\left(\sin\theta \cos\varphi ,\sin\theta \sin\varphi ,\cos\theta \right)$ that reads $\gamma =acos\left(n\cdot n^{\prime }\right)=acos\left[\cos\left(\theta \right)\cos\left(\theta^{\prime }\right)+\right.\left.\sin\left(\theta \right)\sin\left(\theta^{\prime }\right)\cos\left(\varphi -\varphi^{\prime }\right)\right]$ [90]. The induced multipole moments (up to electric quadrupole term) within the meta-atom can then be directly derived based on different combinations of *l* as follows [90]

(14) $$\begin{array}{cc} D & =\int j_{0}\left(k_{0}r^{\prime }\right)P_{\text{in}}\text{d}r^{\prime }+\frac{k_{d}^{2}}{10}\int \frac{15j_{2}\left(k_{d}r^{\prime }\right)}{{\left(k_{d}r^{\prime }\right)}^{2}}\\ & \;\times \left[\left(r^{\prime }\cdot P_{\text{in}}\right)r^{\prime }-\frac{1}{3}r^{\prime 2}P_{\text{in}}\right]dr^{\prime },\\ m & =-\frac{3}{2}i\omega \int \frac{j_{1}\left(k_{d}r^{\prime }\right)}{k_{d}r^{\prime }}\left[r^{\prime }\times P_{\text{in}}\right]dr^{\prime },\\ \hat{M} & =-5i\omega \int \frac{j_{2}\left(k_{d}r^{\prime }\right)}{{\left(k_{d}r^{\prime }\right)}^{2}}\left(\left[r^{\prime }\times P_{\text{in}}\right]⊗r^{\prime }+r^{\prime }⊗\left[r^{\prime }\times P_{\text{in}}\right]\right)\text{d}r^{\prime }\\ \hat{Q} & =\int \frac{3j_{1}\left(k_{d}r^{\prime }\right)}{k_{d}r^{\prime }}\left[3\left(r^{\prime }⊗P_{\text{in}}+P_{\text{in}}⊗r^{\prime }\right)-2\left(r^{\prime }\cdot P_{\text{in}}\right)\bar{\bar{I}}\right]\text{d}r^{\prime }\\ & \;+6k_{d}^{2}\int \frac{j_{3}\left(k_{d}r^{\prime }\right)}{{\left(k_{d}r^{\prime }\right)}^{3}}\left[5r^{\prime }⊗r^{\prime }\left(r^{\prime }\cdot P_{\text{in}}\right)\right.\\ & \;\left.-r^{\prime 2}\left(P_{\text{in}}⊗r^{\prime }+r^{\prime }⊗P_{\text{in}}\right)-\left(P_{\text{in}}\cdot r^{\prime }\right)r^{\prime 2}\bar{\bar{I}}\right]\text{d}r^{\prime } \end{array}$$

where ***D*** corresponds to the exact total electric dipole (TED), ***m*** is the MD moment, and $\hat{Q}$ , and $\hat{M}$ represent the EQ, and MQ tensors respectively. The operators of ⋅, ×, and ⊗ represent the scalar, vector, and tensor products, respectively. The expressions given by Eq. (14) are known as exact multipole moments and are valid for meta-atoms of arbitrary shape regardless of their size and topology. The LWA multipole moments can be derived from the exact expressions of Eq. (14) using small argument approximation (*k*_*d*_ *r*’ ≪ 1) of the spherical Bessel functions $j_{0}\left(k_{d}r\right)\approx 1-{\left(k_{d}r\right)}^{2}/6$ , $j_{1}\left(k_{d}r\right)\approx k_{d}r/3$ , and $j_{2}\left(k_{d}r\right)\approx {\left(k_{d}r\right)}^{2}/15$ as

(15) $$\begin{array}{c} D_{\text{LWA}}=\int P_{\text{in}}dr^{\prime }+\frac{k_{d}^{2}}{10}\int \left\{\left[r^{\prime }\cdot P_{\text{in}}\right]r^{\prime }-2r^{\prime 2}P_{\text{in}}\right\}\text{d}r^{\prime }\\ m_{\text{LWA}}=-\frac{i\omega }{2}\int \left[r^{\prime }\times P_{\text{in}}\right]dr^{\prime }\\ {\hat{M}}_{\text{LWA}}=\frac{\omega }{3i}\int \left(\left[r^{\prime }\times P_{\text{in}}\right]⊗r^{\prime }+r^{\prime }⊗\left[r^{\prime }\times P_{in}\right]\right)dr^{\prime }\\ {\hat{Q}}_{\text{LWA}}=\int \left[3\left(r^{\prime }⊗P_{\text{in}}+P_{\text{in}}⊗r^{\prime }\right)-2\left(r^{\prime }\cdot P_{\text{in}}\right)\bar{\bar{I}}\right]\text{d}r^{\prime } \end{array}$$

where the second term in ***D***_LWA_ is known as the electric toroidal dipole (TD) moment that can interfere with its conventional basic counterpart (first term) either constructively or destructively, leading to a wide spectrum of exotic phenomena such as the formation of anapole state [86]. Using these notations, the far-field scattered power can be readily related to the scattered fields of Eq. (13) using a time-averaged Poynting vector defined as $dP_{\text{Sct}}=0.5\sqrt{\varepsilon_{0}/\mu_{0}}{\left|E_{\text{Sct}}\right|}^{2}r^{2}\text{d}\Omega$ , where dΩ = sin *θ*d*θ*d*φ* represents the solid angle. Therefore, the scattering cross-section, defined as *σ*_Sct_ = *P*_Sct_/*I*_0_, with *I*_0_ being the maximum beam intensity in a focal plane, can be written as follows:

(16) $$\begin{array}{ll} \sigma_{\text{Sct}} & \approx \frac{k_{0}^{4}}{12\pi \varepsilon_{0}^{2}\eta_{0}I_{0}}{\left|D\right|}^{2}+\frac{k_{0}^{4}\mu_{0}}{12\pi \varepsilon_{0}\eta_{0}I_{0}}{\left|m\right|}^{2}+\frac{k_{0}^{6}}{1440\pi \varepsilon_{0}^{2}\eta_{0}I_{0}}\\ & \;\times \underset{x_{1},x_{2}}{\sum }{\left|Q_{x_{1}x_{2}}\right|}^{2}+\frac{k_{0}^{6}\mu_{0}}{160\pi \varepsilon_{0}\eta_{0}I_{0}}\underset{x_{1},x_{2}}{\sum }{\left|M_{x_{1}x_{2}}\right|}^{2} \end{array}$$

wherein $x_{1}=\left\{x,y,z\right\}$ , and *x*_2_ = {*x*, *y*, *z*}. This expression for *σ*_Sct_ is at the heart of studying light–matter interaction within the context of the multipole decomposition approach. In the case of meta-atoms whose dimensions are much smaller than the operating wavelength, the scattering cross-section obtained from the conventional LWA expressions (given by Eq. (15)), agrees well with that calculated using Eq. (14). However, once the dimensions of the meta-atoms become comparable to the operating wavelength, the LWA expressions fail to accurately describe the optical response of the meta-atoms, and the exact expressions should be used. In order to compare the exact and LWA results, in Figure 6 we have plotted the scattering cross-section of two cubic and cylindrical silicon-based meta-atoms whose dimensions are *H* = *w* = 260 nm and *H* = 260 nm and *R* = 169 nm, respectively. It can be seen that while the predicted spectral positions of the induced moments as well as their types (i.e., ED, MD, etc.) calculated using these two approaches agree well, their corresponding amplitudes differ. This difference can be attributed to the discrepancies between the exact and LWA expressions for the multipole moments.

**Figure 6:**
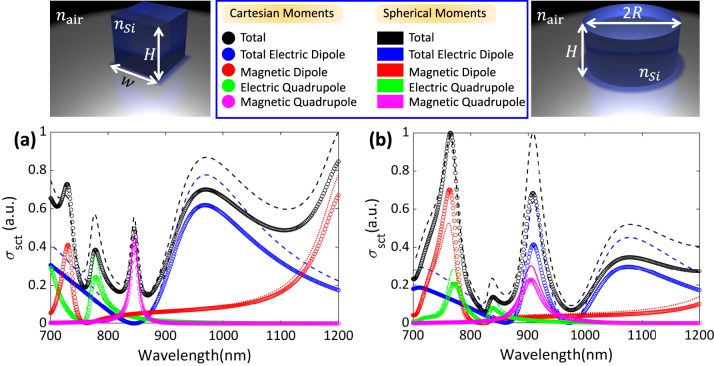
The calculated scattering cross-sections and contribution of exact and LWA moments for (a) cubic and (b) cylindrical subwavelength meta-atoms. The dashed lines correspond to the exact multipolar moments, while the circles represent the LWA moments.

## 4 Mie-tronics in a Nutshell

**Figure 7:**
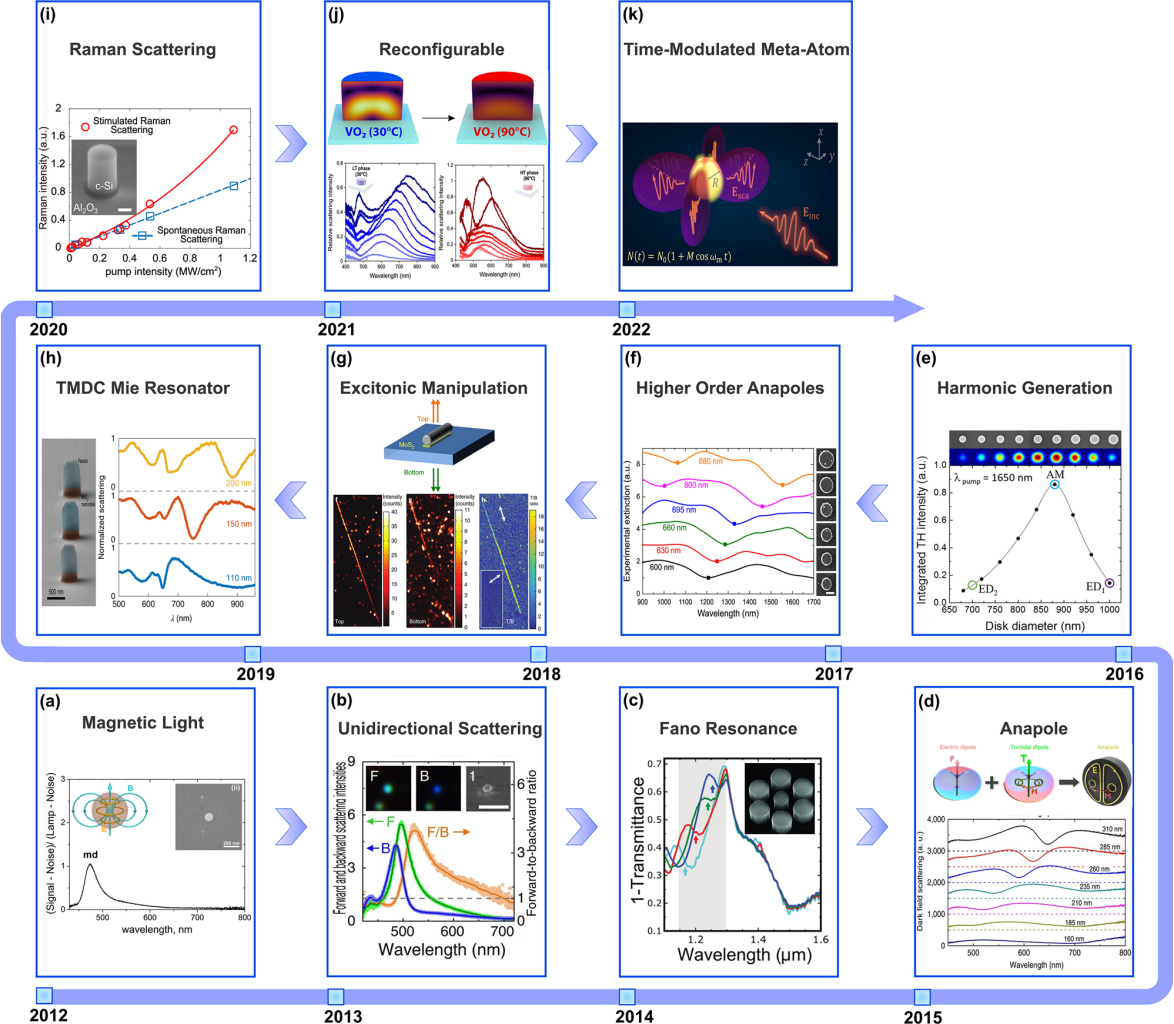
An overview of Mie-tronics covering the traditional light interaction with isolated optical scatterers. (a) Kuznetsov et al. experimentally demonstrated that a spherical silicon nanoparticle can support a strong magnetic dipole resonance at various wavelengths depending on its relative size [102]. (b) Fu et al. experimentally demonstrated that by tuning the size of a spherical silicon nanoparticle, its corresponding far-field radiation pattern can be manipulated such that directional light scattering with a high forward-to-backward scattering ratio is obtained [115]. (c) Chong et al. experimentally demonstrated light scattering by all-dielectric oligomers composed of silicon nanoparticles and the possibility of the excitation of Fano resonances within these configurations [116]. (d) Miroshnichenko et al. demonstrated the existence of non-radiating anapole moments in optics [117]. (e) Grinblat et al. studied the third-harmonic generation from low-loss subwavelength germanium particles supporting anapole moment [118]. (f) Zenin et al. experimentally demonstrated the efficient excitation of higher-order anapole moments within all-dielectric silicon particles that can exceed the first-order anapole state [119]. (g) Cihan et al. experimentally demonstrated that by utilizing a silicon nanowire supporting Mie resonances, one can vary the directionality, polarization state, and spectral emission of two-dimensional materials [120]. (h) Verre et al. showed that nanostructures made of transition metal dichalcogenides can support various multipolar moments such as anapole states [121]. (i) Zograf et al. demonstrated the stimulated Raman scattering for isolated crystalline silicon nanoparticles and experimentally observed a transition from spontaneous to stimulated scattering [122]. (j) Kepic et al. demonstrated that vanadium dioxide nanoantennas can be utilized for designing tunable metasurfaces in the visible range thanks to the phase transition properties of VO_2_ [123]. (k) Asadchy et al. described light scattering by a spherical particle whose permittivity is modulated in time and presented a route to obtain directional light amplification [124].

While the interactions between the conventional Gaussian light beams and nanostructured materials, or metamaterials, have been extensively studied in the last decade [36], [37], [38, 95], [96], [97], in this section, we will briefly review this rapidly developing field research and highlight some unique regimes of such light–matter interactions, and outline their potential applications. While early studies in this field of research focused on the interaction of light with plasmonic nanoantennas [98–100], high refractive index engineered dielectric meta-atoms, have been shown to provide an alternative route to manipulate light through the excitation of their resonant modes, or Mie resonances discussed in the previous section. Such all-dielectric meta-atoms offer the significant advantage of being virtually lossless in the visible and/or near-infrared wavelength ranges, where their plasmonic counterparts suffer from ohmic losses. Moreover, dielectric meta-atoms support both electric and magnetic resonances determined by their topology and material properties (i.e., dielectric constant or refractive index) [101]. In particular, magnetic dipole resonance was proposed and demonstrated in 2012 by Kuznetsov et al. [102] in the visible regime as shown in Figure 7(a). Following this initial demonstration of magnetic resonances in a nonmagnetic, dielectric material, significant effort has been devoted to the studies of the effect of geometries and material properties on such resonances [103–114].

In addition to the excitation of various electric and magnetic type resonances, the interference and interplay between different Mie-type resonances have been shown to enable many remarkable spectral properties of all-dielectric structures [115, 125–129]. In particular, in 2013, Fu et al. have experimentally shown that silicon spherical particles can support strong unidirectional radiation patterns that rely on the interference of electric and magnetic resonances excited within the nanoparticles as shown in Figure 7(b) [115]. Apart from single scatterers, clusters of subwavelength particles of different geometries including dimers, trimers, quadrumers, and hexamers, have been shown to support new regimes of light–matter interactions [116, 130–141]. For instance, in 2014, Chong et al. have both theoretically and experimentally demonstrated the excitation of Fano resonances within an all-dielectric cylindrical heptamer that form due to the interference of the Mie-type magnetic mode of the central meta-atom with the collective resonant mode of the other meta-atoms within the configuration as shown in Figure 7(c) [116]. While a majority of the studies in the field of Mie-tronics before 2013 have been based on the assumption that *any alternating current distribution should radiate electromagnetic energy to the far field*, significant efforts have been devoted to finding non-radiating sources, which were theoretically predicted by Afanasiev and Stepanovsky in 1995 as the destructive interference between electric and toroid dipole moments [142]. Moreover, Zheludev et al. have experimentally shown such a peculiar nonradiating state for the first time in the microwave regime, which was then termed as anapole (from Greek “ana”, “without”, thus meaning “without poles”) [143]. Followed by such a breakthrough, in 2015, Miroshnichenko et al. demonstrated that anapoles can be also realized in the visible region based on the destructive interference between the basic and toroidal contribution of the electric dipole in the far field as shown in Figure 7(d) [117]. Noteworthy that such anapole states are characterized by significant confinement of energy within the optical scatterer, making it a promising platform for enhanced light–matter interactions on the nanoscale that may be particularly important for nonlinear optics applications such as harmonics generation and wavelength conversion as shown in Figure 7(e) [118]. More details on the topic of anapole state and its higher-order counterparts (shown in Figure 7(f)) can be found in [119, 144–162].

Recently, a variety of high-index materials including germanium (Ge), silicon (Si), and titanium dioxide (TiO_2_) has been used to explore various phenomena in the field of Mie-tronics [163]. However, besides these conventional dielectrics, there is a plethora of other materials, such as transition metal dichalcogenides (TMDCs) that exhibit attractive characteristics in the visible and infrared (IR) spectral regions [164–166]. In particular, TMDC crystals are two-dimensional (2D) layers configured in stacked arrangements and are weakly adhered by van de Waals interactions [167]. Each consisting layer comprises three atomic layers, represented as MX_2_, wherein X denotes a chalcogen atom (e.g., X = S, Se, Te) and M indicates a transition metal atom (e.g., M = Mo, W) and can be synthesis by various methods such as molecular beam epitaxy (MBE) [168] and chemical vapor deposition (CVD) [169]. Aside from the numerous applications enabled by these 2D materials, their integration with structured matters supporting Mie-type resonances leads to hybrid entities, which can potentially open a plethora of applications in nano-photonic such as the manipulation of excitonic emission in near- and far-field regimes, routing valley polarized chiral emission, and establishing strong coupling regime of interaction [170, 171], to name just a few. For instance, Cihan et al. experimentally demonstrated that the integration of a silicon nanowire supporting Mie resonances with a MoS_2_-TMDC can change the directionality, polarization state, and spectral emission of the integrated 2D material on demand as it is shown in Figure 7(g) [120]. Recently, it has been revealed that bulk TMDCs can provide an unusually high refractive index in both near-infrared and visible regimes [121, 170, 172, 173], making them favorable candidates for optical applications. Moreover, due to rapid advancements in nanofabrication techniques, it is now possible to create desired patterns directly from bulk TMDCs. In this context, Verre et al. experimentally demonstrated that such TMDC-based nanoparticles can support Mie-type resonances, and the induced resonant modes can interfere with one another to excite nonradiating anapole states as shown in Figure 7(h) [121].

Besides the mentioned applications of Mie-tronics, in recent years several studies have been dedicated to tailoring spontaneous Raman scattering with Mie-like resonances provided by subwavelength particles of various shapes and materials [174]. In this perspective, Zograf et al. [122] observed Stimulated Raman scattering from isolated subwavelength crystalline silicon (c-Si) nanoparticles, for the first time as shown in Figure 7(i). In particular, by optimizing the corresponding dimensions of the nanoparticle and substrate, they have been able to enhance the stimulated emission without overheating, which can provide an additional degree of freedom for obtaining Raman scattering from nanoscale structures. However, despite the wide spectrum of applications and great potential enabled by subwavelength scatterers, a grand challenge facing these platforms is that their response is set in stone after they have been designed and cannot be changed afterward. Therefore, an immense effort has been made to overcome this limitation of passive isolated meta-atoms by exploiting mechanical, thermal, and electrical tuning mechanisms such that the scattering response of the meta-atoms can be controlled in real-time by control over external stimuli (such as strain, temperature or voltage) rather than the change in the geometry [123, 175–189]. An example of such efforts is illustrated in Figure 7(j), where Kepic et al. [123] experimentally demonstrated that vanadium dioxide (VO_2_) meta-atoms can be tuned using temperature and operate as optical switches changing their properties from dielectric to plasmonics. In addition, more recently, time-modulated platforms have emerged as a new class of active devices in which the external stimuli controlling the optical properties are varying periodically in time [190–201]. Such temporally varying structures have been shown to enable a wide range of novel physical phenomena including nonreciprocity [202–208], and signal amplification [124, 209, 210], as shown in Figure 7(k). One of the most remarkable properties of such temporally modulated structures is the possibility of frequency mixing enabling the generation of higher-order frequency harmonics without the need for high-intensity pump beams [190–192].

Finally, in addition to the studies of resonant excitations of individual meta-atoms, one- and two-dimensional periodic arrangements of these meta-atoms, or meta-chain and metasurfaces, have been also shown to facilitate beam steering [211], holography [212], nonlinear harmonic generation [213–216], Kerker, anti-Kerker, and transverse Kerker effects [112, 217, 218], invisibility [219–221], absorber [222–225], optical force manipulation [226–227], and topological waveguiding [230] to name a few. However, as this review aims to address the interaction of structured lights with structured matters and elucidate the potential avenues opened by their synergy, we limit our discussion to the case of single scatterers.

## 5 Structured-light- structured-matter interaction

In recent years, a significant effort has been put to generate and implement structured light for a wide range of applications including optical trapping, metrology, probing, and data processing [49, 50, 64], while in parallel, the field of Mie-tronics continued to develop rapidly, opening new avenues for shaping and manipulation of light [36–47]. The interaction of structured light with structured matter is expected to give rise to a wide range of light–matter interactions that are not accessible using conventional Gaussian beams (or plane waves) or nonresonant Rayleigh scatterers. In this section, we focus on structured light-structured matter interactions and their experimental studies and outline their potential applications.

### 5.1 Interactions of SAM- and OAM-carrying light beams with matter

**Figure 8:**
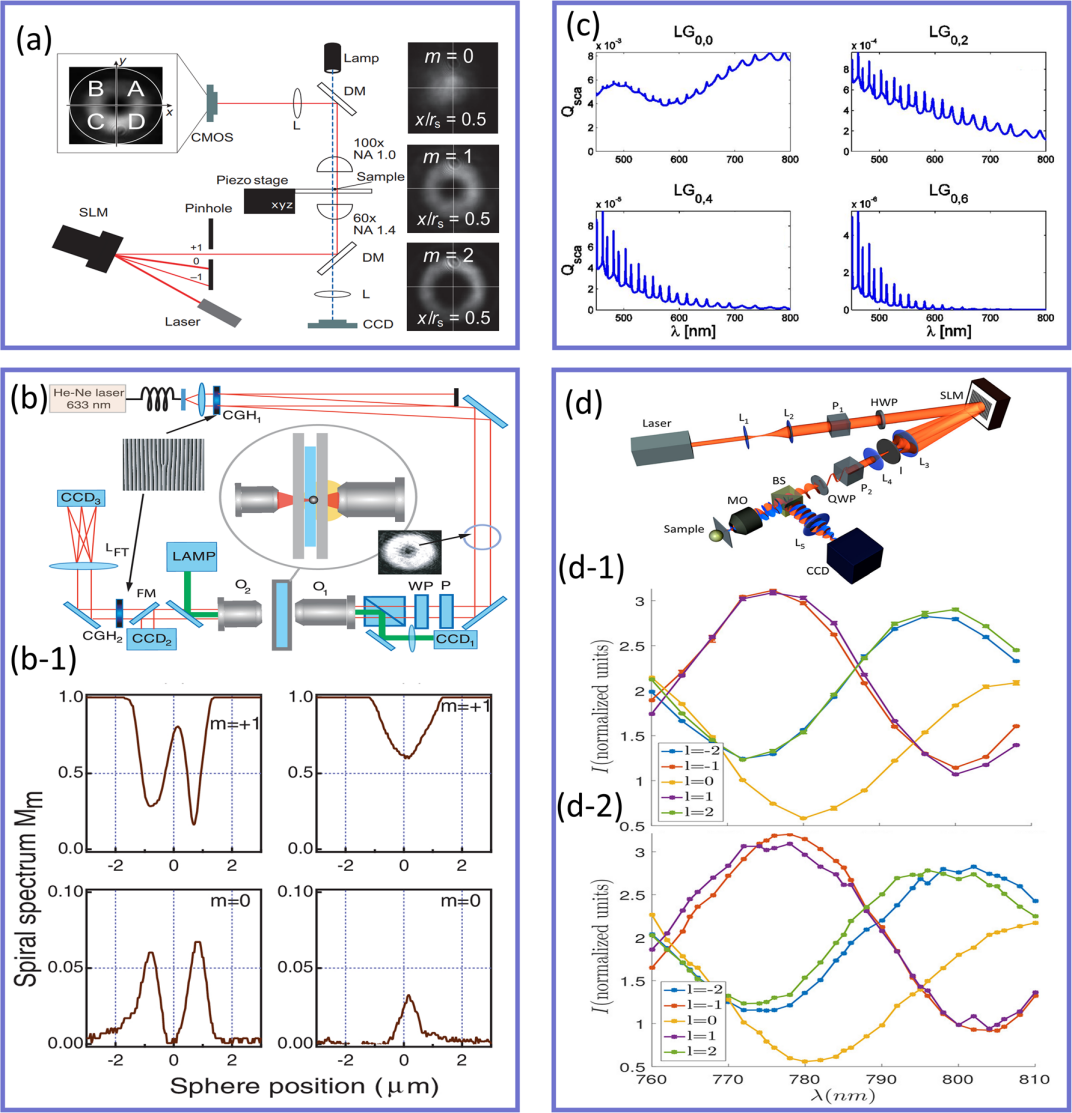
The role of angular momentum of light in scattering from subwavelength meta-atoms. (a) The experimental setup used by Garbin et al. [231] to distinguish the topological charge of an optical vortex from the measured Mie scattering spectra. The inset demonstrates the scattered intensity distributions of a displaced off-axis spherical particle for Gaussian (*m* = 0) and LG (*m* = 1 and *m* = 2) illuminations. Upon illumination of a Gaussian beam, the measured intensity distribution moves parallel to the direction of translation, while for an LG illumination, the fringes move along a diagonal plane. (b) The experimental investigation carried out by Petrov et al. [237] to characterize the geometrical properties of spherical particles from their interactions with beams carrying angular momentum. (b-1) The dependence of the optical response of the scatterers under the illumination with the beams with two different topological charges of *m* = 0 and *m* = 1 as a function of the spherical meta-atom position. The first and second columns correspond to a particle whose radius is 2 μm and 1 μm, respectively. (c) The calculated scattering efficiency of a spherical particle as a function of wavelength for various mode indices of LG beams, $\left(n_{\text{LG}},m_{\text{LG}}\right)$ , shown on top of each plot [238]. (d) The experimental setup used by Zambrana-Puyalto et al. [239] aimed at exploring the role of SAM and OAM on the scattering spectra of spherical meta-atoms. The back-scattered light beams measured upon the interaction of LG beams with the SAM of (d-1) +*ℏ* and (d-2) −*ℏ* and topological charges −2 < *m* < +2 as a function of wavelength.

In most general case, light can possess both spin and orbital angular momentum. The spin angular momentum (SAM) is related to the polarization state of the light. In this context, each photon carries an angular momentum of ±*ℏ*, where the ± sign indicates whether the polarization is right-handed or left-handed. In addition to the SAM, light beams with an azimuthal phase dependence of $\exp\left(-im\varphi \right)$ carries an orbital angular momentum (OAM) that takes values of *mℏ*, where *m* is an integer number known as the topological charge (TC) or OAM mode number, that determines the number of intertwined helical phase fronts in the light beam [70]. Both SAM and OAM contribute to a light beam’s total angular momentum and can be used to change the optical response of subwavelength particles. In this context, the scattered field resulting from the interaction of an LG beam carrying a TC of *m* with a spherical particle has been shown to be sensitive to the modulus and sign of the incident beam topological charge [231] as illustrated in Figure 8(a). In these experiments, the TC of the incident light beam was changed from *m* = 1 to *m* = 8 and the size of the spherical particles was set to be comparable with the beam waist as *R*_*s*_ = 0.5 μm. Specifically, a linearly polarized Gaussian beam at the wavelength of *λ* = 1.064 μm was converted to an LG beam using an SLM and then focused onto the spherical particles by a 60× objective lens with the numerical aperture (NA) of 1.4. The forward scattered light from the particles was subsequently collected using a 100× objective lens with an NA of 1. The primary objective of this paper was to investigate whether the analysis of the scattered field, without directly observing the movement of an optically trapped particle due to the transfer of angular momentum, would enable the distinction of the sign of the phase dislocation. For this purpose, the authors considered the scenario in which a sphere is displaced from the optical axis along the *x*-direction. In this situation, the sphere experiences an angular phase gradient, which exhibits opposite signs depending on whether the topological charge is positive or negative. The inset of panel (a) demonstrates the captured forward scattered light intensities for three values of topological charge ranging from *m* = 0 to *m* = 2. For a Gaussian beam with the TC of *m* = 0, the distribution of the measured intensity moves in the direction of particle translation, while when a beam with a non-zero TC was used, it moved at an angle with respect to the direction of displacement. Such differences have been attributed to the phase distribution of the LG beam. These results can be potentially used for position detection of dielectric particles using structured light beams carrying an OAM [232, 233]. It should be noted that under tight focusing conditions, particularly with a high NA of 1.4, the longitudinal component of the electric field becomes comparable to the transverse field, and thus, the field interacting with the scatterer is no longer within the paraxial domain [234–236]. As a result of employing tightly focused beams beyond the paraxial approximation, the observation of spin–orbit coupling becomes possible, as demonstrated in other studies [23–237]. However, this aspect was not addressed in the present paper. In a similar study, the scattering data of LG beam interaction with nanospheres of various sizes were used by Petrov et al. [237] to determine the geometrical properties of the scatterers, such as their size, position, and ellipticity, as well as the refractive index contrast between the particle and the host medium as shown in Figure 8(b). In particular, a linearly polarized He–Ne laser beam with the operating wavelength of *λ* = 633 nm was sent to a computer-generated blazed hologram (CGH_1_) to obtain LG beams with various topological charges of *m* = 0 and *m* = 1 and beam sizes of 1.5 μm. A CHG is a hologram whose pattern is designed and calculated using computer algorithms to create a specific, optimized diffraction pattern. The term “computer-generated” is used because the precise control and optimization of the hologram’s properties, such as the direction and efficiency of the diffracted light, are achieved through digital calculations and design, enabling the creation of custom holograms tailored for specific applications. The resulting LG beams were focused onto the spherical samples having diameters ranging from 1 μm to 20 μm and the total scattered fields (scattered from spherical particles and the nonscattered part of the incoming beam) were collected. Figure 8(b-1) demonstrates the output spectrum corresponding to the two spheres with the diameter of 2 μm (first column) and 1 μm (second column) with respect to their spatial position when the incident light beam carried TC of *m* = 1 (first row), and *m* = 0 (second row). As was predicted and demonstrated in panel (a), for the same spherical particle (e.g., first column), when the TC of the light beam changes, its output spectrum differs due to the difference between the phase distributions of Gaussian and LG beams. In addition, when the sphere is displaced by half its diameter (second column), its corresponding output spectra for the same TC change drastically, providing an opportunity to evaluate the size of nanoparticles from their scattering spectra. Indeed, Zambrana-Puyalto et al. [238, 239], have shown that the LG beam can be used as another degree of freedom in manipulating the scattering spectra of dielectric spherical particles (besides the size, and refractive index of the particle and the host medium) as it is shown in Figure 8(c). Particularly, this figure demonstrates the scattering cross-section of the 1.3 μm dielectric sphere for four different LG mode indices of $\left(n_{\text{LG}},m_{\text{LG}}\right)=\left[\left(0,0\right),\left(0,2\right),\left(0,4\right),\left(0,6\right)\right]$ . As was predicted in [231], when the azimuthal mode index changes from *l* = 0 to *l* ≠ 0, the corresponding optical response of the particle changes significantly, while for larger values of the azimuthal index, the emerged ripples in the scattering spectra, aroused due to excitation of higher order moments, increase. Experimental studies of the effect of the angular momentum and the polarization handedness of the incoming light beam on the optical response of spherical particles are shown in Figure 8(d). The experimental setup used for this purpose resembles the ones shown in panels (a) and (b), with the notable difference being the implementation of a tunable laser beam, offering operating wavelengths between 760 and 810 nm, to probe the TiO2 meta-atoms, as opposed to a fixed-wavelength laser utilized in the setups of panels (a) and (b). In this setup, the incident beam is first polarized using a linear polarizer and a half-wave plate (HWP) to align with the polarization axis of the SLM, which imparts the desired phase onto the incoming light beam. A combination of lenses L3 and L4, along with an iris (I), is employed to select the first diffracted order and expand it to match the size of the high NA = 1.1 microscope objective (MO). Subsequently, a quarter-wave plate (QWP) is utilized to modify the polarization of the light beam, which is then tightly focused onto the sample using the same MO that collects the backscattered light. Figure 8(d-1) shows the measured backscattering spectra of the fabricated samples using light beams having a topological charge in the range of −2 < *m* < 2. These experimental studies confirmed the theoretical predictions in that the measured backscattering spectra show oscillating behavior for all the incident vortex beams. For instance, while the Gaussian beam possesses a dip at *λ* = 780 nm, its optical response alters significantly when the angular momentum of the incoming light changes from zero to ±*ℏ* or ±2*ℏ* per photon. However, when the polarization handedness of the incident light varies, the measured spectra exhibit the same spectral behavior with comparable peak magnitudes as shown in Figure 8(d-2). These results (panels (c) and (d)) provide deeper insight into the field of light–matter interaction and elucidate the role of angular momentum and polarization handedness of light as alternative mechanisms to selectively excite a particular multipolar moment and manipulate its spectral position, which can potentially be used in several scattering phenomena such as spectroscopy, cytometry or dark-field microscopy [231, 237], [238], [239] to mention a few.

Besides the studies of structured light beams’ interactions with structured matters shown in Figure 8, many other remarkable results have been reported in recent years [240–254]. For instance, the interaction of nanoholes with a vortex beam carrying both SAM and OAM is studied in [240] demonstrating that in contrast to the total angular momentum, the polarization handedness of light may not be preserved in the process of spin–orbit coupling. Moreover, a significant effort has also focused on the studies of the selective excitation of multipolar resonances [247] and optical manipulations [242] of all-dielectric nanostructures interacting with various types of structured light beams. We also note that besides the dielectric platform, the interactions between structured light and plasmonic resonators have attracted considerable interest in recent years due to their potential applications in various fields, such as sensing, and imaging [255]. For instance, recently, it has been demonstrated that plasmonic nanoantennas can be used as an alternative mechanism to directly measure the information encoded in twisted lights via converting the OAM into spectral information using bright and dark modes [253].

### 5.2 Interaction of cylindrical vector beams with structured media

**Figure 9:**
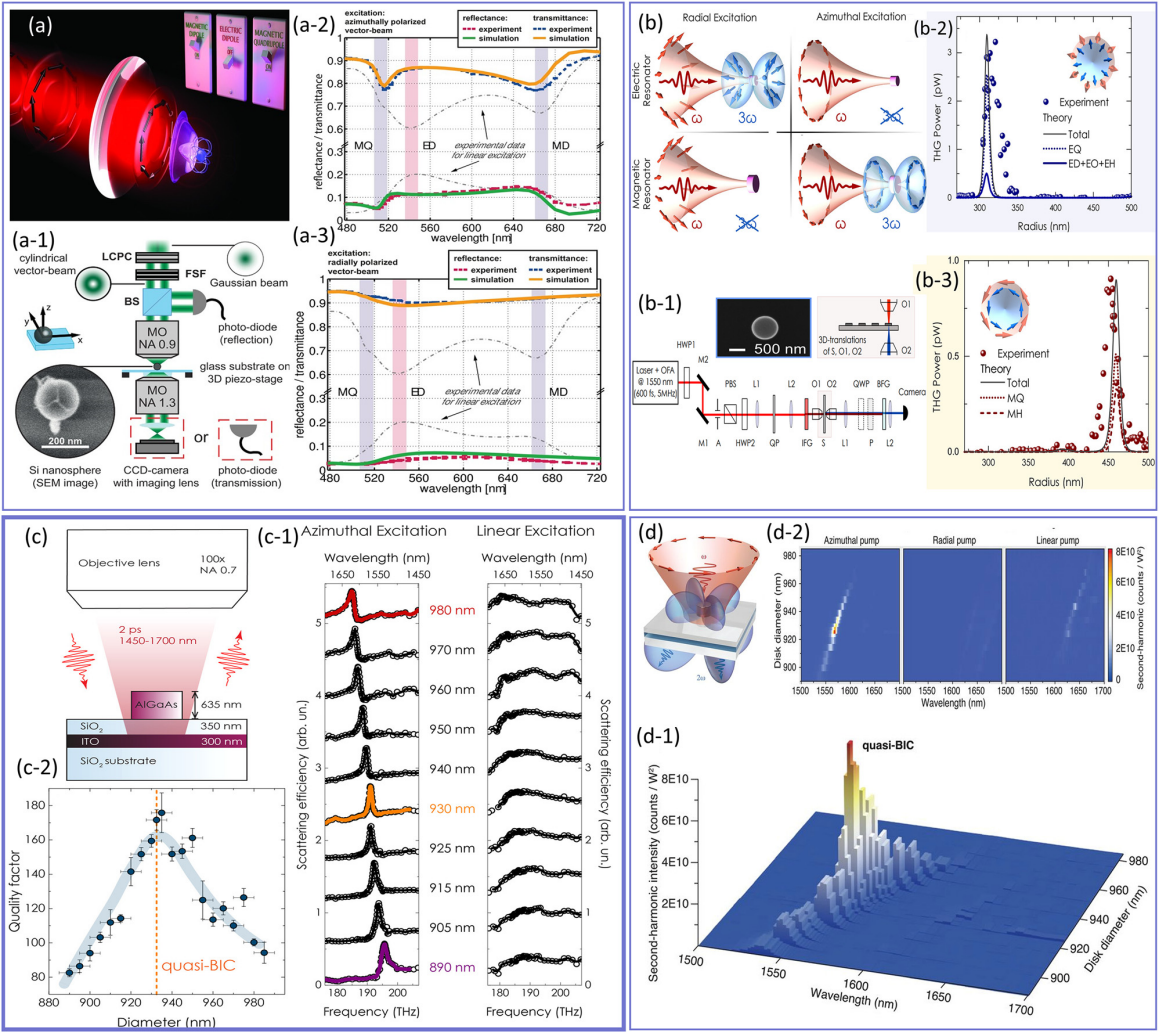
The potential avenues opened by CVB interaction with meta-atoms. (a) The schematic illustration of selective excitation of multipolar moments within the subwavelength dielectric particle. (a-1) The experimental setup utilized by Wozniak et al. for studying the interaction of a CVB with an isolated nanosphere [256]. The measured transmittance and reflectance of the isolated particle under (a-2) azimuthally and (a-3) radially polarized light beams. Upon the change in the structure of the incoming light beam, the induced Mie-type resonant mode within the subwavelength scatterer varies. (b) The artistic demonstration of selective excitation of Mie resonances for nonlinear enhancement application. The first meta-atom supports EQ under RP excitation and generates TH, whereas efficient TH is generated in the second meta-atom due to the excitation of MQ under APB [257]. (b-1) The setup used for experimental investigation carried out by Melik-Gaykazyan et al. to measure the TH efficiency under (b-2) RPB and (b-3) APB. (c) The schematic demonstration of the excitation setup used in [258] to investigate the origin of QBIC in terms of Fano resonances. (c-1) The measured reflectance spectra of the subwavelength scatterer as a function of its diameter for azimuthal (first column) and linear (second column) polarizations, respectively. (c-2) The calculated results of the Q-factor extracted from the measured reflectance of the panel (c-1). (d) The graphic demonstration of the SHG in a cylindrical subwavelength meta-atom under APB excitation [259]. (d-1) The three-dimensional measurement result of the SH intensity under APB illumination as a function of the nanodisk dimension and pump wavelength. (d-2) The intensity enhancement map measured for three scenarios of APB (first column), RPB (second column), and linear (third column) excitations.

In addition to the interaction of OAM and SAM carrying light beams with structured media, several studies were dedicated to the investigation of vector beams’ interactions with optical nanostructures. Importantly, it was shown that the light beams themselves can be engineered to excite new resonances in nanoscale particles. For example, Wozniak et al. [256] demonstrated that by tailoring the spatial structure of the incident light beam, particularly using a tightly focused APB and RPB with a high NA = 0.9 objective lens, individual multipole resonances can be selectively excited while other multipoles are simultaneously suppressed, as it is schematically shown in Figure 9(a). To experimentally demonstrate such new regimes of light–matter interactions, well-separated individual silicon nanospheres were fabricated on top of a glass substrate and investigated using the setup shown in Figure 9(a-1). The reflectance and transmittance are shown in Figure 9(a-2) and (a-3), for APB and RPB, respectively. As can be seen from these two panels, upon changing the polarization of the incident beams, the scattering spectra of the same particle vary significantly, and new resonances emerge. For instance, upon the illumination with the APB, both MD and MQ resonances emerge within the spectrum of the particle, while when the polarization is changed to the RPB, the previously excited resonances (MD and MQ) disappear, and new ED resonance emerges. Therefore, by choosing a tightly focused CVB, the desired multipolar moment can be selectively excited and manipulated, which allows for the identification of resonances in terms of their type and orientation.

While an optical response of a nanoresonator is largely determined by its geometry and material, the efficiency of light coupling to a particular resonant mode strongly depends on the structure of the excitation beam. Recently, structured light-induced Mie resonances have been exploited in the context of nonlinear optics as shown in Figure 9(b). In particular, Melik-Gaykazyan et al. [257] utilized APB and RPB for the excitation of the resonances of the silicon nanocylinder. The meta-atom dimensions were chosen such that once the structure of the incident light beam changes from radially polarized to azimuthally polarized beam, the optical response of the particles switches from EQ to MQ as shown in panel (b). The optical response of the cylindrical meta-atom with a height fixed to 700 nm, and a radius varying between 270 and 500 nm was investigated under RPB and APB excitation at the pump wavelength of *λ*_p_ = 1550 nm as shown in Figure 9(b-1). The linear optical response of the meta-atom indicates that for the case of RPB excitation, the nanocylinder with the radius of 310 nm (first meta-atom) possesses a strong EQ response while when the incoming light beam changes to APB, this resonant peak disappears. On the other hand, under the APB excitation of the particle with a radius of 460 nm (second meta-atom), the optical response is dominated by the MQ resonant mode. To experimentally demonstrate such a capability of CVB interaction with structured matter, an experimental setup of Figure 9(b-1) was utilized. In a different approach from previous works, Kruk et al. [65] obtained radially polarized and azimuthally polarized light beams using a fabricated metasurface. The laser beam at the fundamental frequency of 193.55 THz illuminated the sample and its third harmonics (TH) counterpart was collected as shown in Figure 9(b-2) and (b-3) for RPB and APB, respectively. Both the simulation and experimental results indicate that the APB light beam enhances the TH in the close vicinity of the MQ resonance, while for the RPB excitation, dominant TH power is observed near the EQ resonance. These results demonstrate the possibility of using structured light as another degree of freedom in tailoring the optical response of subwavelength dielectric nanoparticles in nonlinear applications.

Up to this point, we focused on the bright modes that efficiently radiate into free space. In contrast, dark modes, such as the bound state in the continuum (BIC), are confined to the structure with infinite lifetime and zero radiation. In general, BICs are a unique class of resonant states in which the state is perfectly discrete and spatially confined in space but can exist at the same energy as a continuum of states propagating to the infinity [260]. Subsequently, BIC resonant modes do not couple to the continuum of radiation modes outside the structure and are “trapped” in the structure. In quantum mechanics, momentum and position are related through the uncertainty principle, Δ*x*Δ*p* ≥ *ℏ*/2, indicating that a particle’s momentum and position cannot be simultaneously known with complete precision. In this context, the confinement of the bound state to a finite region in real space results in the delocalization of the system’s properties in momentum space. In other words, from a momentum-space perspective, a BIC resonant mode can be seen as anomalies where the BIC energy is equal to the energy of the continuum radiation modes. In contrast, other resonant states, such as Fabry–Perot modes or surface plasmon polaritons, do not exhibit such anomalies in momentum space. These modes have finite lifetimes and can couple to the radiation continuum outside the structure, leading to a broadening of the resonance peak in momentum space. While ideal BICs are inaccessible from external excitation, a quasi-BIC (QBIC) can potentially be accessed from the free space either under the oblique incidence or upon breaking the in-plane symmetry under normal incidence (symmetry-protected BIC), providing a platform for achieving high-quality factor (Q-factor) resonances and boosting light–matter interaction at the nanoscale [261]. It is noteworthy that while most of the studies of QBIC resonances were limited to plane wave interactions, recently, several studies focused on the potential applications of azimuthally polarized light beams in the efficient coupling of light to the QBIC resonant modes [258, 259, 262, 263]. Very similar to BIC resonant modes, Fano resonances represent a universal wave phenomenon having two essential features of asymmetric and ultrasharp spectral line shapes, which are attributed to the interference of a continuum of bright modes with the dark modes [264]. In 2021, Melik-Gaykazyan et al. [258] experimentally uncovered the underlying physical mechanism linking the physics of QBIC and the asymmetric features of Fano resonances in the isolated aluminum gallium arsenide (AlGaAs) nanodisks of various diameters and explained how the transition between these two resonant modes can be obtained, as shown in Figure 9(c). The measurement results for various size nanocylinders in the spectral range of 1450 nm–1700 nm for two types of polarizations, that is azimuthal (first column) and linear (second column) are shown in Figure 9(c-1). As can be seen from the first column, an asymmetric resonant line shape emerged in the scattering spectrum of the nanocylinder, whereas for the linear polarization (second column), the induced resonant peak is suppressed regardless of the nanocylinder size. Importantly, for the azimuthal polarization, depending on the size of the meta-atoms, the sign of the Fano parameter changes from negative (red curve for 980 nm diameter) to positive (purple curve for 890 nm diameter). In this process, the Fano resonance transitions to the QBIC resonant mode at the meta-atom diameter of 930 nm (orange curve) where the asymmetrical line shape changes to a symmetrical one as shown in Figure 9(c-2). The obtained results of panel (c) highlight the role of the pump beam structuring to enable selective excitation of high-*Q* modes that may find applications for the enhancement of nonlinear conversion efficiency.

As we discussed earlier, azimuthally polarized light beams provide unique opportunities to excite QBIC resonant modes within subwavelength particles with high *Q*-factors. Recently, the interaction of APB with the isolated AlGaAs nanocylinders was studied by Koshelev et al. [259], who demonstrated the possibility to boost the second-harmonic generation (SHG) via inducing QBIC resonant modes with a *Q*-factor of 190 (Figure 9(d)). In particular, the proposed configuration is a three-layer heterostructure consisting of a silica substrate, indium tin oxide (ITO), and an AlGaAs resonator supporting a Mie resonance at the second harmonic (SH) wavelength simultaneously with the fundamental frequency. Two orders of magnitude enhancement of the SH intensity in the close vicinity of the QBIC resonance under the illumination of APB is shown in Figure 9(d-1). For comparison, the SHG intensity maps for other types of illuminations (RPB and linear polarization) are provided in Figure 9(d-2), clearly revealing the difference in the enhancement of the SHG between the APB excitation and other kinds of beams. It should be noted that the advances of structured light–matter interactions in the context of nonlinear optics are not limited to the aforementioned works and many other remarkable results have been reported, including the possibility of selective excitation of magnetic multipolar moments in isolated cylinders for boosting the SHG or the enhancement of the THG from all-dielectric oligomers [265–273].

### 5.3 From radiative to nonradiative states with structured lights

**Figure 10:**
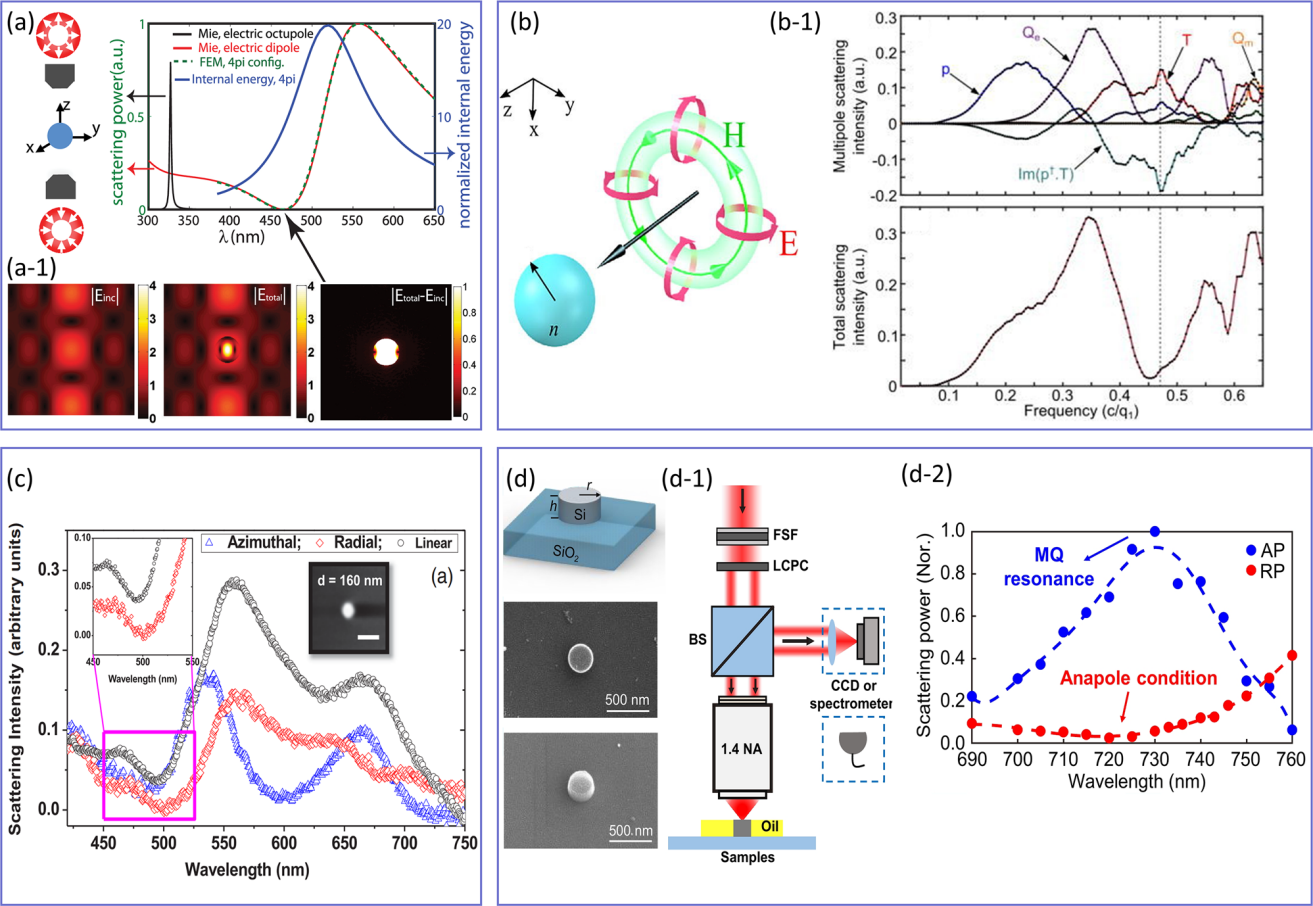
Formation of non-radiating anapole state under different types of structured light incidences. (a) The 4*π* illumination setup utilized by Wei et al. in Ref [275] to suppress the radiation to the far field (left side) and the results of numerical simulations corresponding to the normalized internal energy and scattering power spectrum (right side). (a-1) The calculated incident (left column), total (middle column), and scattered (right column) electric field distributions at the operating wavelength corresponding to the anapole excitation. (b) The artistic illustration of a TM FD pulse impinging on a dielectric spherical meta-atom [276]. (b-1) The numerical calculation of LWA moments excited within the nanoresonator of radius *R* = *q*_1_ (top row) and its corresponding scattering intensity. (c) The measurement results of the scattering spectra of isolated silicon-based nanospheres (the SEM image shown in the inset) under the illumination of linear (black curve), radial (red curve), and azimuthal (blue curve) polarization light beams [277]. (d) The diagram of a Si nanodisk fabricated on a glass substrate and its SEM images utilized in Ref [278] to experimentally investigate the formation of two distinct radiative and non-radiative resonant modes, under the excitation with CVB. (d-1) The optical setup utilized by Lu et al. for measuring the backscattering spectra of the fabricated sample and its (d-2) measured spectra for APB (blue color graph) and RPB (red color curve), respectively.

So far, we focused on the discussion of isolated multipolar moments. Here we show that the interference between induced multipolar moments leads to a plethora of fascinating light–matter interactions at the nanoscale. In particular, the theory of electromagnetic multipole expansion, including charge-current spherical and Cartesian decompositions, is important for the theoretical description of these interactions [88–94]. For instance, it allows us to describe a toroidal moments family, which can be represented as the poloidal currents flowing along the meridians of a torus for the electric toroidal dipole (ETD) moment and more complex current distributions for higher-order toroidal moments, also known as mean square radii [274]. In particular, an electric dipole anapole (EDA) state forms upon the destructive interference between an electric dipole (ED) and its toroidal counterpart (i.e., ETD), which subsequently leads to vanishing scattering accompanied by strong energy confinement within the subwavelength scatterer [161]. The studies of the anapole states in engineered meta-atoms have received considerable attention due to their potential applications in strong exciton coupling, second and third-harmonic generation, Raman scattering, photocatalysis, guiding energy, and lasing [160–162]. However, despite the fruitful progress in this field of research, the complete suppression of scattering from the EDA state is prohibited, due to the simultaneous excitation of MQ modes within the presented meta-atoms. To overcome such a problem, Wei et al. [275] carried out a theoretical investigation to excite an ideal radiationless anapole state within an isotropic high-index dielectric nanosphere via a “4*π* configuration” comprising of two counter-propagating radially polarized light beams with the same amplitude and a *π* phase difference, as shown in Figure 10(a). In particular, owing to the presence of longitudinally polarized electric field $\left(E_{z}\right)$ and zero magnetic fields at the focal point of the tightly focused RPB, the magnetic moments within the resonator are expected to be suppressed, yielding the excitation of an ideal nonradiating anapole state. Figure 10(a) demonstrates the scattering cross-section and normalized internal energy of the spherical meta-atom under the 4*π* illumination. As can be seen, at the wavelength where the anapole condition is satisfied (***P***_LWA_ = −*ik* ***T***_LWA_), that is *λ* = 464 nm, the scattering power becomes nearly zero, which is also accompanied by an unperturbed nearfield shown in the first column of Figure 10(a-1). However, despite such a zero-scattering power, the calculated energy within the nanoresonator volume was reported to be eight times higher compared to the scenario wherein the meta-atom is absent. Such energy confinement has also been verified in the nearfield distribution, as shown in Figure 10(a-1). Another class of nonradiative solutions of Maxwell’s equations that recently attracted significant interest are so-called flying doughnut (FD) pulses modulated in both space and time. Theoretically proposed over 20 years ago, flying doughnuts are one-cycle pulses, having a toroidal structure in space. Recently, Zheludev and colleagues proposed and experimentally demonstrated the possibility of conversion of an ordinary light pulse into a flying doughnut by using a polarization converter and a carefully designed metasurface to manipulate both the spatial and spectral structure of the input pulse [21]. They have shown that FDs show many unique properties including non-trivial field transformations upon reflection from interfaces and the excitation of toroidal response and anapole modes in the matter, thus offering new opportunities for telecommunications, sensing, and spectroscopy [21, 86]. Based on the similarity between the topological structure of FD pulses and toroidal dipolar excitation (shown in Figure 10(b)), Raybould et al. [276] have recently demonstrated the excitation of strong toroidal mode and anapole state upon the interaction of FD pulses with dielectric spherical particles. A transverse magnetic (TM) FD pulse with its radial ( $E_{\rho }^{\text{TM}}$ ) and longitudinal ( $E_{z}^{textTM}$ ) electric fields, as well as its azimuthal magnetic field ( $H_{\theta }^{\text{TM}}$ ), can be written as

(17) $$\begin{array}{c} E_{\rho }^{\text{TM}}=4if_{0}\sqrt{\frac{\mu_{0}}{\varepsilon_{0}}}\frac{\rho \left(q_{2}-q_{1}-2iz\right)}{{\left[\rho^{2}+\left(q_{1}+i\tau \right)\left(q_{2}-i\sigma \right)\right]}^{3}}\\ E_{z}^{\text{TM}}=-4f_{0}\sqrt{\frac{\mu_{0}}{\varepsilon_{0}}}\frac{\rho^{2}-\left(q_{1}+i\tau \right)\left(q_{2}-i\sigma \right)}{{\left[\rho^{2}+\left(q_{1}+i\tau \right)\left(q_{2}-i\sigma \right)\right]}^{3}}\\ H_{\theta }^{\text{TM}}=-4if_{0}\frac{\rho \left(q_{1}+q_{2}-2ict\right)}{{\left[\rho^{2}+\left(q_{1}+i\tau \right)\left(q_{2}-i\sigma \right)\right]}^{3}} \end{array}$$

where *τ* = *z* − *ct*, *σ* = *z* + *ct*, and *f*_0_ represent an arbitrary normalization constant, while *q*_1_ and *q*_2_ denote the effective wavelength of the pulse and the focal region depth, respectively. The results of numerical simulations shown in the first row of Figure 10(b-1) suggest that while for the particular spectral position of *ν* = 0.46*c*/*q*_1_ (marked by black dashed line), the ED (blue line) and TD (red line) possess resonant peaks, the EQ moment has a minimum. However, despite the presence of such electrical resonant modes, the total scattering intensity spectrum given in the second row of the panel (b-1) closely resembles the EQ moment. This similarity is attributed to the large value of the interference term between electric and toroidal dipoles, given by $Im\left(P_{\text{LWA}}^{†}\cdot T_{\text{LWA}}\right)$ , that results in suppression of their total scattering intensity. It should be mentioned that using FD pulses, as opposed to plane wave excitation and the implementation of two tightly focused counter-propagating beams, allowed the excitation of the anapole states without inducing higher multipoles with a single illumination source.

Despite the recent theoretical and experimental advancements in the field of anapole state with plane wave illuminations, the experimental demonstration of these nonradiating resonant modes with structured lights is still in its infancy. To this end, Parker et al. [277] have recently illustrated the excitation of a non-scattering anapole state in all-dielectric silicon-based spherical particles with various sizes, using tightly focused radially and azimuthally polarized CVBs as shown in Figure 10(c). In particular, the experimental studies of particles with a diameter of 160 nm, indicate that upon RPB illumination, the scattering spectra of the meta-atom possess pronounced minima in contrast with the APB and linear polarization excitation. This result can be explained by the destructive interference of electrical and toroidal dipole moments. Followed by such an experimental demonstration, and the ability of CVBs to selectively excite Mie resonant modes, very recently, Lu et al. [278] have experimentally demonstrated the transition from nonradiating anapole state to radiative MQ resonant mode within a cylindrical meta-atom (the top and side views of scanning electron microscopy (SEM) images of the fabricated samples are shown in Figure 10(d)) using radially and azimuthally light beams, respectively, as illustrated in Figure 10(d-1).

The schematic of the experimental setup is shown in Figure 10 (d-1). Figure 10 (d-2) shows a resonant peak at 735 nm under APB illumination, which is attributed to MQ resonant mode (blue curve), while when the polarization was changed to RPB (red curve), the scattering spectrum possesses a dip at the same spectral position corresponding to the excitation of an anapole state. These results offer new opportunities for switching between the maximum and minimum scattering at the same wavelength by utilizing tightly focused AP and RP beams.

### 5.4 Structured light-induced chiroptical response

**Figure 11:**
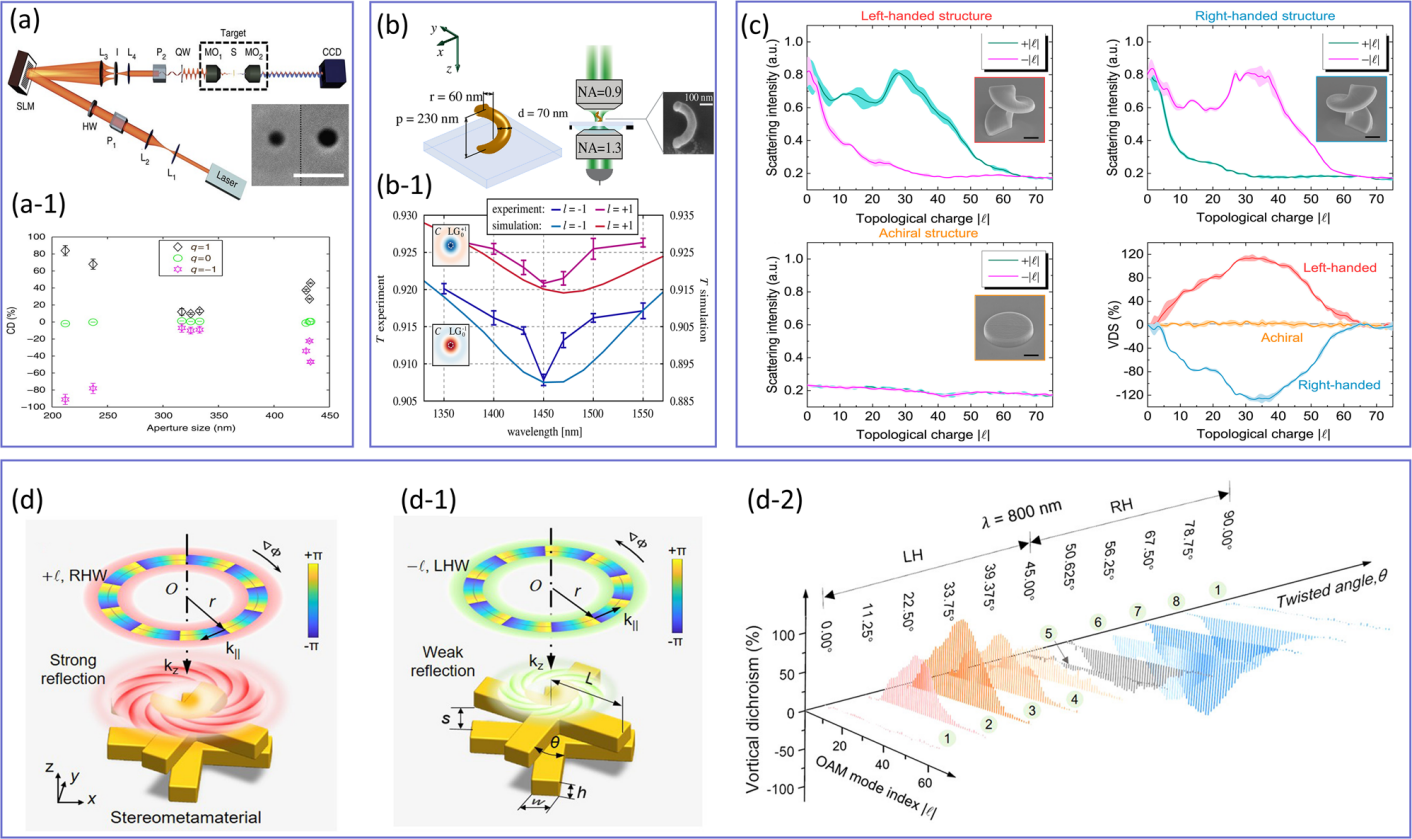
The potential applications of structured lights in chiral light–matter interactions. (a) The experimental setup utilized by Zambrana-Puyalto in Ref [281] for investigating the role of angular momentum of the incident light in the CD of a non-chiral nanostructure shown in the inset. (a-1) The measurements result of the CD(%), calculated from $\text{C}{\text{D}}_{m}\left(\%\right)=\left[\left(I_{m}^{L}-I_{m}^{R}\right)/\left(I_{m}^{L}+I_{m}^{R}\right)\right]\times 100$ , for three different TC of $m=\left[-1,0,+1\right]$ as a function of the nano-holes diameters. While the green curve is associated with the TC of *m* = 0, the black and pink color graphs correspond to *m* = 1 and *m* = −1, respectively. (b) The artistic illustration of the chiral structure and its SEM image used by Wozniak et al. in Ref [282] for probing the vorticity of the incident beam and its (b-1) measured spectra for various incident topological charges as a function of the operating wavelength. (c) The experimental results associated with the scattering intensity of the left-handed (top left), right-handed (top right), and achiral (bottom left) microstructures illuminated by a linearly polarized light beam carrying an orbital angular momentum of *mℏ* [283]. The corresponding VD of the measured results are also shown in the bottom right figure with red (for left-handed meta-atom), blue (for right-handed scatterer), and orange (achiral structure) color curves. (d) and (d-1) The conceptual sketch of tunable VD based on the twisted stereometamaterials proposed by Liu et al. in Ref [284] and its (d-2) corresponding experimental results as a function of the incident beam topological charge and the twisted angle of *θ*.

Chiral objects, including the DNA and proteins, are structures whose mirror images (enantiomers) are not superimposable with that of their original topologies. They can possess different chiroptical responses, such as optical rotatory dispersion (ORD), and circular dichroism (CD) [279, 280]. Circular dichroism is defined as the differential absorption of left and right circular polarization (LCP or RCP). While traditionally the CD is associated with chiral geometries, Zambrana-Puyalto et al. [281] have experimentally shown the possibility of inducing chiroptical responses from achiral objects using light beams carrying an OAM as shown in Figure 11(a). The experimental setup and the process of generating OAM carrying light beam are the same as that of Figure 8 with the high NA = 1.1 objective lens, yet the achiral samples are gold-based circular apertures with various diameters (the SEM image is shown in the inset) that are centered with respect to the incident beam. The CD of such a sample is measured in a two-step process, such that first the transmitted intensity of the LCP light beam carrying the topological charge of *m* (i.e., $I_{m}^{L}$ ) is measured for each diameter. Next, the same measurement was repeated for the RCP (i.e., $I_{m}^{R}$ ). The normalized CD was then calculated as $\text{C}{\text{D}}_{m}\left(\%\right)=\left[\left(I_{m}^{L}-I_{m}^{R}\right)/\left(I_{m}^{L}+I_{m}^{R}\right)\right]\times 100$ and measured for three TC of $m=\left[-1,0,1\right]$ as illustrated in Figure 11(a) with respect to the aperture size. As can be seen from this figure, for the symmetric light beam (*m* = 0) normally incident on the symmetric sample, the measured CD is zero as expected. However, once the topological charge of the incoming beam acquires nonzero values of *m* = ±1, its corresponding phase distribution becomes asymmetric, and a giant CD arises. Indeed, the mirror symmetry of such an achiral system is broken due to the inherent asymmetrical features of the incident structured light beam. Following this experimental study, in 2019, Wozniak et al. [282] explored the role of OAM interaction with a plasmonic chiral scatterer (shown in Figure 11(b)). A chiral subwavelength helical scatterer shown in the inset of Figure 11(b) was illuminated by a tightly focused LG beam (the focusing objective lens has NA of 0.9) carrying OAM of ±*ℏ* and their corresponding transmittances were measured with respect to wavelength as shown in Figure 11(b-1). The measured spectrum clearly illustrates the fact that once the sign of the topological charge changes, the obtained transmittance from the chiral meta-atom also changes in such a way that it can be used to distinguish the OAM of the incoming light beam from its optical chirality defined as $C=-\left(\omega /2c^{2}\right)Im\left[E^{*}\cdot H\right]$ . Following these studies, OAM or vortex beams were further utilized to determine the chirality of structure, which led to the new concept of vortical dichroism (VD).

Ni et al. [283] have both theoretically and experimentally illustrated a significant chiroptical response arising from the interaction of a non-paraxial OAM-carrying light beam (focused with NA of 0.9) with intrinsically chiral microstructures as it is illustrated in Figure 11(c). As can be seen from the reported results of this work, for intrinsically chiral structures possessing both left- and right-handiness (see panels (c-1) and (c-2)), the scattering spectra exhibit different chiroptical responses as a function of the topological charge of the incoming beam, while for the achiral sample (panel (c-2)) no prominent change is observed. By defining the VD in a similar fashion as that of Zambrana-Puyalto’s work, (i.e., $\text{VD}=\left[2\left(I_{+\left|m\right|}-I_{-\left|m\right|}\right)/\left(I_{+\left|m\right|}+I_{-\left|m\right|}\right)\right]\times 100$ with $I_{\pm \left|m\right|}$ denoting the scattering intensity corresponding to the TC of $\left|m\right|$ ), the VD scattering reaching the peak value of 120% was experimentally achieved for chiral nanostructures as shown in Figure 11(c-2).

Finally, in 2022 Liu et al. [284] proposed a twisted stereo-metamaterial, shown in Figure 11(d), that can lead to a tunable VD ranging from −97% to +98%. In particular, it was shown that regardless of the sign and value of the topological charge of the incoming wave, when the twisted angle (*θ*) is zero (achiral structure), the handiness of the incident beam does not affect the chiroptical response of the meta-atom (Figure 11(d)), whereas different behavior can be observed once the twisted angle is changed to nonzero values (chiral geometry shown in Figure 11(d-1)), providing an opportunity to achieve tailorable response upon engineering the rotational angle. Experimentally measured VD spectra with respect to the rotational angle and the value of the topological charge at the fixed wavelength of 800 nm are shown in Figure 11 (d-2). As can be seen from this figure, for the achiral structure (*θ* = 0°), the measured VD is zero regardless of the value of TC, whereas once the level of geometrical achirality increases, the VD spectrum reaches the maximum value of +98% for *θ* = 22.5° and then drops to the minimum value of −97% for *θ* = 67.5°, confirming the tailorable chiroptical response resulting from the structured light–matter interaction. As the final remark, it is worth noting that in the context of structured light, both paraxial and non-paraxial solutions have been demonstrated to enable chiral light–matter interactions [279–284]. In the paraxial approximation, the transverse components of the wave vector are significantly smaller than the longitudinal component, resulting in light propagating primarily along the optical axis. On the other hand, non-paraxial light beams do not adhere to these assumptions, giving rise to more intricate and diverse phenomena, such as vortical dichroism.

## 6 Summary and outlook

In this paper, we have outlined the recent advances in the field of structured light–matter interactions and elucidated the role of the spatial structure of light beams in manipulating the scattering spectra of dielectric meta-atoms and their assemblies. We overviewed various theoretical and experimental approaches to study the synergy of structured light and structured matter in linear, nonlinear, and chiral optics. In this outlook section, we attempt to highlight the remaining open questions, potential trends, and future directions in this fascinating branch of modern optics, which we named “*singular optics empowered by engineered optical materials*”.

Despite significant progress in the field of structured light interaction with resonant Mie particles, the majority of the studies focused on spherically or cylindrically symmetric particles, while the synergy of structured light beams and complex-shaped meta-atoms has not been fully explored. One possible research direction would be the study of the interaction of an OAM-carrying light beam, with nonspherical and randomly tilted meta-atoms with varying aspect ratios. For instance, owing to the particular structure of the LG beam and its OAM, such an interaction with arbitrary shape meta-atoms can lead to entirely new scattering characteristics and degrees of freedom for selective excitation, suppression, and manipulation of individual resonant modes as compared to those obtained with a conventional Gaussian beam and spherically/cylindrically symmetric particles, which in turn opens new prospects for applications such as remote sensing and communications in scattering media.

Moreover, electric anapole state, which arises due to the destructive interferences of electric and toroidal electric dipoles, has been introduced as the fundamental class of non-radiating sources and has enabled a plethora of applications ranging from nonlinear optics to thermodynamics. Nevertheless, from the standpoint of physics and duality of Maxwell equations, the existence of other types of anapole states such as magnetic anapole is also possible and can potentially provide enhanced radiation suppression and confinement of electromagnetic energy, if they are spectrally overlapped with their electrical counterparts. While the existence of magnetic anapole with structured light has been theoretically investigated in [285], the possibility of exciting hybrid anapoles is yet to be addressed. Therefore, another research direction would be the study and the design of meta-atoms that support these new non-radiative states, called hybrid anapoles, which are formed due to the destructive interference between electric and magnetic basic and toroidal multipoles of various types.

Another interesting scattering phenomenon not discussed here in detail is super-scattering, which is the phenomenon opposite of a non-radiating state. In particular, energy conservation enforces a limit on the portion of the energy scattered into one scattering channel. As a result, the maximum contribution of scattering cross-section (SCR) from any given multipole moment is limited to $\left(2l+1\right)\lambda^{2}/2\pi$ , wherein *l* represents the order of the multipole [286, 287]. However, it has been shown that the SCR of subwavelength particles can exceed such a single-channel limit if at least two multipole resonances spectrally overlapped with one another. As was discussed earlier, the structure of the incident light can directly affect the optical response of the meta-atom and leads to either spectrally overlapping or decoupling of the contributing moments. Therefore, one possible future research direction would be the use of structured light to selectively change the optical response of the meta-atom from non-radiating to super-scattering states, such that two opposite optical responses are obtained on a single platform.

Finally, as we discussed in this review, optical metasurfaces offer several exciting opportunities for the generation and manipulation of structured light including the OAM-carrying beams, CVBs, and FD pulses. However, engineered nanostructures may revolutionize many other branches of singular optics, including the generation of STOVs, higher-dimensional structured light (such optical links, knots, etc.), and linear and nonlinear quantum optics with structured light for atom trapping, communications, and computing. Therefore, we expect that the combination of singular optics with Mie-tronics will continue broadening the horizons of nanophotonics and offer new perspectives for meta-optics, leading to novel applications beyond our current imagination.
